# Alternative therapeutic approaches for combating multi-drug-resistant bacteria: Reverse vaccinology against *Enterobacter cloacae*^☆^

**DOI:** 10.1016/j.jgeb.2025.100519

**Published:** 2025-06-17

**Authors:** Gabriela Guerrera Soares, Marcelo Silva Folhas Damas, Pedro Mendes Laprega, Rebecca Elizabeth Shilling, Eduarda Oliva Ribeiro Rangel, Louise Teixeira Cerdeira, Murillo Rodrigo Petrucelli Homem, André Pitondo-Silva, Andrea Soares da Costa-Fuentes, Maria-Cristina da Silva Pranchevicius

**Affiliations:** a Departamento de Genética e Evolução, Universidade Federal de São Carlos, São Carlos, SP, Brazil; b Department of Vector Biology, Liverpool School of Tropical Medicine, Liverpool, United Kingdom; c Departamento de Computação, Universidade Federal de São Carlos, São Carlos, SP, Brazil; d Programa de Pós-graduação em Odontologia e Tecnologia Ambiental, Universidade de Ribeirão Preto, Ribeirão Preto, Brazil

**Keywords:** *Enterobacter cloacae*, Multi-epitope vaccine, Reverse vaccinology, Subtractive proteomics

## Abstract

*Enterobacter cloacae* is a clinically significant opportunistic and multidrug-resistant bacterium that causes a range of hospital-acquired infections, particularly in intensive care units. However, studies on vaccine development have been limited, and no vaccine currently protects against *E. cloacae*. Here, we employed subtractive proteomics, reverse vaccinology, and immunoinformatic approaches to design a multi-epitope-based vaccine targeting *E. cloacae*. Analysis of 21 complete *E. cloacae* genomes associated with human infections revealed 1,352 proteins linked to essentiality, resistance, and/or virulence, 39 of which were non-human and non-gut homologs. From this refined selection, 9 were found to be antigenic, extracellular, or exported to the outer membrane and used to construct 4 multi-epitope vaccines (VEC1-4) containing antigenic (threshold of ≥0.5), non-allergenic, conserved, hydrophilic (GRAVY < 0), exposed, and non-toxic epitopes. They were all processed and presented through the MHC class pathway, while also showing high population coverage. VEC1 showed the most consistent performance, with the highest average binding affinity (−24.07 kcal/mol), docking score (−322.21), and the most favorable dissociation constant at 37 °C. VEC1 was shown to be conformationally stable, with a secondary structure predominantly made up of alpha-helices and coils. The *in silico* analysis suggested that VEC1 can be efficiently expressed in an *E. coli* system, and it is currently awaiting *in vivo* testing to confirm its precise efficacy, safety, and immunogenicity. These findings provide valuable insights for developing novel approaches to prevent and control the spread of multidrug-resistant bacteria.

## 1 Introduction

Antimicrobial resistance (AMR) has emerged as one of the most significant global public health challenges of the current century. Over 39 million individuals globally are predicted to die due to antibiotic-resistant infections within the next 25 years.^1^ The World Health Organization has published a list of priority bacterial pathogens requiring the development of novel strategies to effectively combat antimicrobial resistance due to their severe clinical consequences, treatment complexity, and high transmissibility.^2^ Enterobacteriaceae, including *Enterobacter* spp., are among the priority pathogens highlighted by WHO.^3^, ^4^

*Enterobacter cloacae* is a Gram-negative bacterium in the *Enterobacterales* family that resides in the environment and in the commensal enteric flora of healthy humans.^5^, ^6^ Over the past decades, *E. cloacae* has emerged as a common opportunistic nosocomial pathogen, capable of causing a wide range of infections, including peritonitis,^7^ endophthalmitis,^8^ brain abscesses,^9^ urinary tract infections,^10^, ^11^ spondylodiscitis,^12^ endocarditis,^13^ and meningitis.^14^

*E. cloacae* frequently displays resistance to a wide range of antibiotics, including the majority of β-lactams,^15^ quinolones,^16^ sulphonamides,^17^ and colistin.^18^ AmpC β-lactamase produced by *E. cloacae* contributes especially to carbapenem resistance when combined with the expression of extended-spectrum β-lactamases (ESBLs), reduced outer membrane permeability, and efflux pump activity.^19^ Following *Escherichia coli* and *Klebsiella pneumoniae*, carbapenem-resistant *E. cloacae* (CREC) is the third most prevalent carbapenem-resistant *Enterobacteriaceae* species responsible for nosocomial infections.^20^, ^21^ CREC is now a significant threat to global public health and is a high priority for preventive treatment development.^22^

Vaccines can be highly effective tools for combating antimicrobial resistance, as they help reduce the number of infections, decrease antibiotic use, and so slow the emergence and spread of resistance.^23^ Reverse vaccinology, a revolutionary *in silico* approach, identifies vaccine candidates from the genomes of drug-resistant pathogens.^24^ This strategy is made possible by advancements in genome sequencing, genomic data availability, and bioinformatics tools and was first successfully applied to *Neisseria meningitidis* group B.^25^, ^26^, ^27^ It has since been employed against other pathogenic agents, including *Neisseria gonorrhoeae*,^28^*Staphylococcus cornubiensis* NW1T,^29^*Naegleria fowleri*,^30^ Monkeypox virus (MPXV)^,^^31^*Klebsiella pneumoniae*,^32^*Pseudomonas aeruginosa*,^33^ the SARS-CoV-2 coronavirus,^34^, ^35^ *Mycobacterium tuberculosis*^36^^,^*Staphylococcus* spp*.*,^37^, ^38^ *Salmonella typhi*, and *Salmonella paratyphi.*^39^

RV quickly identifies specific protein sequences, known as epitopes, which are the most likely to elicit potent immune responses without the traditional need for pathogen growth. The selected epitopes can be combined to develop multi-epitope vaccine candidates to enhance their effectiveness against highly diverse and rapidly evolving antibiotic-resistant strains.^25^, ^28^, ^30^, ^40^

There are currently few preventative strategies and no vaccine against *E. cloacae* despite the high demand for these. This study used RV techniques to identify potential epitopes within the *E. cloacae* proteome and subsequently developed a novel multi-epitope vaccine candidate, VEC1, currently awaiting *in vivo* validation.

## 2 Material and methods

**Fig. 1:**
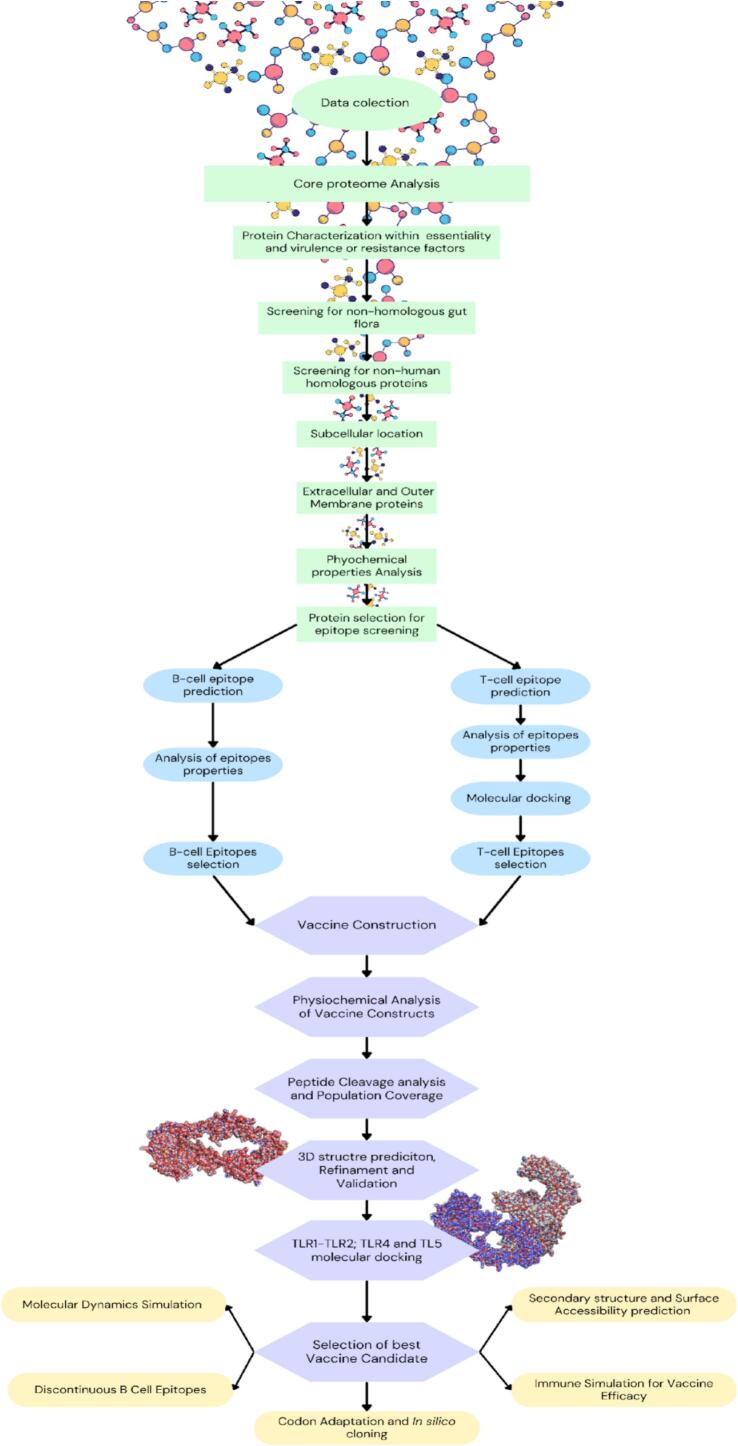
Flowchart illustrating the methodological approach employed to develop a multi-epitope vaccine against *E. cloacae*. Green: subtractive proteomic pipeline (core proteome analysis, pathogenicity filtering, host compatibility tests, and surface protein prediction). Blue: Immunoinformatic screening (MHC-I/II prediction and B-cell linear mapping). Purple: Vaccine engineering (construct assembly, structural modeling, and validation). Yellow: Final validation (immune simulation, *in silico* cloning, codon optimization).

This study employed subtractive proteomics and a reverse vaccinology approach to identify potential vaccine candidates against known *E. cloacae* strains (see Fig. 1 for the methodology).

### 2.1 Data collection and core protein selection

Twenty one complete proteomes from *E. cloacae* strains associated with human infections, including the representative proteome of *E. cloacae* strain 1382 (GCF_905331265.2), were retrieved from the National Center for Biotechnology Information (NCBI) (https://www.ncbi.nlm.nih.gov/genbank/). All these proteomes were submitted to a pan-genome analysis to identify core, accessory, and unique genes. Here, we used the program BPGA version 1.3 with USEARCH as the default protein clustering tool.^41^ A 60 % sequence identity cutoff was used to delineate the core protein set. The strain designation, RefSeq assembly, accession numbers, biosample information, country, and year are detailed in Table S1.

### 2.2 Screening of essential genes and virulence and resistance factors

Essential core proteins were identified using BLASTp searches within the Database of Essential Genes (DEG) (https://tubic.org/deg/public/index.php), a frequently updated repository of essential prokaryotic proteins.^42^, ^43^ Additionally, the putative virulence factors were identified using the Virulence Factor Database (VFDB) (https://www.mgc.ac.cn/VFs/main.htm), a versatile and comprehensive analysis platform for bacterial virulence factors (VFs).^44^

Alongside these screens, BLASTp searches were performed to detect proteins linked to antibiotic resistance within the Comprehensive Antibiotic Resistance Database (https://card.mcmaster.ca/)^45^ and the Antibiotic Resistance Gene-ANNOTation database (https://www.mediterranee-infection.com/acces-ressources/base-de-donnees/arg-annot-2/).^46^ The first covers the entire spectrum of antimicrobial resistance genes^47^ while the second serves as a rapid bioinformatics tool that identifies putative new AR genes in bacterial genomes.^48^ The cutoff values for all BLASTp analyses were a bit-score > 100, e-value ≤ 10^−4^, and similarity >60 %.

### 2.3 Subtraction of gut microbiota homologous and human homologous proteins

Previously identified essential, virulent, and/or resistance proteins were analyzed against a custom database of 79 bacterial proteomes from the gut microbiome using BLASTp inquires.^49^, ^50^ This strategy identified and removed proteins that were homologous to those in the gut microbiome to mitigate against the risk of cross-reactivity with beneficial gut bacteria. A second BLASTp analysis compared the remaining proteins against the *Homo sapiens* reference proteome (GCA_000001405.29) to exclude those that were homologous to human proteins. This analyzes the safety of the vaccine by reducing the likelihood of triggering an autoimmune response by reducing the chance of cross-reactivity and autoimmunity. Both analyses employed an e-value threshold of ≤ 10^−4^ and a similarity cutoff of >60 %.

### 2.4 Prediction of subcellular localization

Two distinct computational methods were employed to determine the subcellular localization of the selected proteins. The first, PSORTb v3.0.2 (https://www.psort.org/psortb/), uses a probabilistic algorithm to analyze the amino acid composition, signal peptides, transmembrane helices, and localization-associated motifs of proteins to determine their subcellular localization.^51^ The second program, the BUSCA server (https://busca.biocomp.unibo.it), identifies signal peptides, GPI anchors, and transmembrane domains to identify the subcellular location of both globular and membrane proteins.^52^, ^53^, ^54^ The results were compared to enhance the accuracy and confidence of the predictions of the subcellular location of these proteins.

### 2.5 Analysis of antigenicity and physicochemical properties of putative vaccine targets

Exposed proteins were subjected to an antigenicity analysis using the VaxiJen v2.0 tool (https://www.ddg-pharmfac.net/vaxijen/VaxiJen/VaxiJen.html),^55^ a widely utilized tool employed to forecast the antigenicity of T cell epitopes. For this analysis, a threshold of ≥0.5 was implemented, as it yields the highest accuracy across most models. Additionally, the online Expasy ProtParam server (https://web.expasy.org/protparam/) was used to calculate the physicochemical properties of all the proteins, including their molecular weight, protein length, and GRAVY value. The GRAVY (Grand Average of Hydropathicity) value is the sum of all hydropathy values of the amino acids divided by the length of the protein sequence. Negative GRAVY values indicate hydrophilic proteins.^56^, ^57^

### 2.6 Transmembrane domains and secretory pathway analysis

The topology of our transmembrane proteins was assessed utilizing the DeepTMHMM server (https://dtu.biolib.com/DeepTMHMM), the most comprehensive and highest-performing method to predict the topology of both alpha-helical and beta-barrel transmembrane proteins.^58^ Additionally, secretory pathways were analyzed using SignalP 6.0 (https://services.healthtech.dtu.dk/services/SignalP-6.0/), a server-based, deep neural network method that can identify the five main categories of signal peptide (SP) sequences.^59^

### 2.7 Screening of T-cells and B-cells epitopes

We used the IEDB Tepitool (https://tools.iedb.org/tepitool/)^60^ and NetCTLpan 1.1 web server (https://services.healthtech.dtu.dk/service.php?NetCTLpan-1.1)^61^ to predict MHC class I peptides considering a set of 27 distinct alleles representing over 97 % of the global population. These computational tools are widely employed in the field to identify peptides that specifically recognize the MHC molecules necessary for T-cell-mediated immune responses.^62^ The NetCTLpan 1.1 server employs sequence-processing methods, such as proteasome cleavage, TAP binding, and MHC-I binding, to accurately identify cytotoxic T-lymphocyte epitopes.^61^ The threshold value for NetCTLpan 1.1 was set at 0.75.^63^ We only considered epitopes with nine amino acids, as this is the most common length of MHC class I peptides in a range of 8–11 resides.^60^

MHC-II epitope prediction was conducted to analyze 26 of the most prevalent human class II alleles from the DP, DQ, and DR loci using the Tepitool server with the IEDB recommended approach.^60^ MHC class II molecules typically bind peptides ranging from 12 to 20 amino acids in length, with a core of about 9 amino acids being the most crucial for binding. Typically, 15 residue peptides are used to optimize these predictions.^60^

The linear B-cell epitopes were predicted with the IEDB and ABCpred servers (https://webs.iiitd.edu.in/raghava/abcpred/),^64^ using the default parameters. B-cell epitopes display structural variability, ranging from 3 to 85 amino acids in length, though continuous epitopes typically fall within a 15 amino acid range.^64^, ^65^

## 3 Immunogenicity, Antigenicity, Toxicity, Allergenicity, GRAVY and IFN-Inducing analysis of selected epitopes

The immunogenicity of MHC-I binding epitopes was evaluated using the IEDB server (https://tools.immuneepitope.org/immunogenicity/),^66^ where higher, more positive scores indicate a greater likelihood of eliciting an immune response. The antigenicity of MHC class I, MHC class II, and B-cell epitopes was analyzed using the VaxiJen v2.0 server, applying a ≥0.5 threshold.^55^ Toxicity predictions were carried out using the default parameter settings of the ToxinPred server (//https://www.imtech.res.in/raghava/toxinpred/index.html).^67^ This server predicts the toxicity of different peptides by using a machine learning approach, specifically support-vector machines (SMV), combined with a quantitative matrix.^68^ Next, the potential allergenicity of the epitopes was evaluated based on the key physicochemical properties of proteins using the AllerTop server (https://www.ddg-pharmfac.net/AllerTOP/feedback.py).^68^ Afterward, the ProtParam server (https://web.expasy.org/protparam/) was utilized to determine the GRAVY values of the epitopes by analyzing the physicochemical properties of proteins.^56^ Finally, the capacity of MHC class II epitopes to stimulate IFN-γ production was assessed using the IFNepitope2 server (https://webs.iiitd.edu.in/raghava/ifnepitope2/).^69^

### 3.1 Prediction of three-dimensional (3D) structure of selected epitopes and molecular docking

The selected MHC class I and MHC class II epitopes were submitted to the PEP-FOLD3 server (https://bioserv.rpbs.univ-paris-diderot.fr/services/PEP-FOLD3/), an online tool that can predict the three-dimensional structures of peptides ranging from 5 to 50 amino acids in length through *de novo* folding techniques.^70^ Docking experiments were then conducted using both the widely utilized ClusPro 2.0 server (https://cluspro.bu.edu/login.php?redir=/home.php)^71^ and the HawkDock server (https://cadd.zju.edu.cn/hawkdock/), which predicts binding structures and identifies key residues involved in protein–protein interactions by generating Molecular Mechanics/Generalized Born Surface Area (MM-GBSA) scores.^72^ The PRODIGY function of the HADDOCK webserver (https://rascar.science.uu.nl/prodigy/) refined the docked complexes generated by ClusPro 2.0 by including ΔG and Kd (M) values at 37 ℃ to produce binding affinity scores.^73^ The MHC class I epitope docking was performed against the following HLA alleles: HLA-A01:01 (PDB: 6AT9), HLA-A02:01 (PDB: 3UTQ), HLA-B15:01 (PDB: 1XR8), HLA-B35:01 (PDB: 1ZSD), HLA-B39:01 (PDB: 4O2E), HLA-B53:01 (PDB: 1A1M), HLA-B58:01 (PDB: 5IM7), HLA-B44:03 (PDB: 1SYS), and HLA-A2.1 (PDB: 1I1Y). The docking of MHC class II epitopes was investigated against a panel of HLA-DRB alleles, comprising HLA-DRB01:01 (PDB: 2FSE), HLA-DRB03:01 (PDB: 1A6A), HLA-DRB104:01 (PDB: 2SEB), HLA-DRB115:01 (PDB: 1BX2), HLA-DRB301:01 (PDB: 2Q6W), HLA-DRB302:02 (PDB: 3C5J), and HLA-DRB501:01 (PDB: 1H15). These alleles were selected for their high global prevalence and extensive coverage of HLA supertypes, and the epitopes were filtered by their ability to bind to multiple HLA molecules, in accordance with established multi-epitope vaccine design strategies.^74^, ^75^, ^76^, ^77^ The epitopes with the highest average binding affinity and MM-GBSA values were then selected for vaccine construction.

### 3.2 Population coverage analysis

The IEDB population coverage analysis tool (https://tools.iedb.org/population/) was used to calculate the populational coverage of all our MHC-I and −II epitopes based on HLA genotypic frequencies obtained from the Allele Frequency Database.^78^ The genotypes covered 16 different geographical areas, encompassing 115 countries and 20 ethnicities, to ensure extensive coverage by the vaccine.

### 3.3 Vaccine construction

**Fig. 2:**
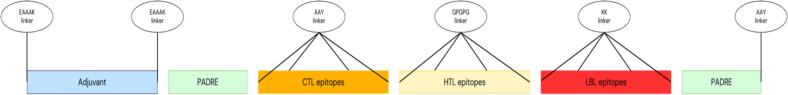
Schematic presentation of the final multi-epitope vaccines against *E. cloacae*. The amino acid sequence contains an adjuvant at the N-terminal, PADRE sequences, as well as CTL, HTL, and LBL epitopes, all connected by the indicated linkers.

The vaccine design involved linking CTL (MHC-I epitopes), HTL (MHC-II epitopes), and BCL (B-cell epitopes) epitopes using three specialized linkers: AAY, GPGPG, and KK. AAY linkers connected the Universal Pan HLA DR sequence (PADRE – AKFVAAWTLKAAA) to the CTL epitopes, as well as among the CTL epitopes themselves. Meanwhile, GPGPG linkers were used to link the CTL epitopes to the HTL epitopes, in addition to the HTL epitopes to each other. Finally, KK linkers were employed to connect the HTL epitopes to the BCL epitopes and BCL epitopes to other BCL epitopes. The adjuvant sequences, the HBHA protein and it’s conserved HBHA sequence,^79^ the 50 s ribosomal protein L7/L12,^80^ and the FliC flagellin,^81^, ^82^ were joined using EAAAK linkers at the N and C termini.^54^ Additionally, EAAAK linkers were utilized to connect the PADRE sequence to four different adjuvant sequences: the HBHA protein, the conserved HBHA sequence, the 50S ribosomal protein L7/L12, or FliC flagellin. These adjuvants increase the vaccine’s potential to stimulate specific immune pathways and give a more robust and targeted immune response.^83^ The schematic of all vaccine constructs is shown in Fig. 2.

### 3.4 Predicting the Antigenicity, allergenicity, solubility, and physicochemical properties of vaccine candidates

The antigenicity of the vaccine candidates was evaluated using VaxiJen v2.0,^54^ while the AllerTOP server (https://www.ddg-pharmfac.net/AllerTOP/)^84^ was used to assess the allergenic potential of all vaccine constructs. The solubility of the vaccine constructs was estimated using SOLpro from the Scratch Protein Predictor (https://scratch.proteomics.ics.uci.edu/) by employing a two-stage SVM architecture based on multiple representations of the primary sequence. Its accuracy exceeds 74 % when ten-fold cross-validation runs are used.^85^

The physicochemical properties of all vaccine candidates were analyzed using the Expasy ProtParam server (https://web.expasy.org/protparam/).^56^ This tool provided information on the number of amino acids, molecular weight, theoretical isoelectric point, instability index, aliphatic index, and GRAVY values.

### 3.5 Prediction of tertiary structure, refinement, and validation of vaccine constructs

The 3D structures of the vaccine constructs were generated using the I-TASSER server (https://zhanggroup.org/I-TASSER/), a leading platform in 3D protein structure prediction based on Critical Assessment of techniques for Structure Prediction (CASP) experiments.^86^ The 3D structures were then further refined using the Galaxy Refine web server (https://galaxy.seoklab.org/refine), which is designed to improve both the global and local quality of the protein structures.^87^

The quality of the refined vaccine constructs was evaluated using a number of tools. SAVES v6.1 (https://saves.mbi.ucla.edu/) assessed the structural quality of protein models. The PROCHECK tool evaluated the stereochemical quality of protein structures, with Ramachandran plots created for each structure.^88^ The ERRAT server provided an overall quality factor for the constructs by analyzing the predicted strength of non-bonded interactions between different atom types.^89^ Finally, the ProSA web server (https://prosa.services.came.sbg.ac.at/prosa.php)^90^ determined the z-scores of the refined vaccine models, comparing them to the experimentally determined z-scores of native proteins.

### 3.6 Protein-protein docking

The selected vaccine candidates were docked against the TLR4-MD2 (PDB:3FXI), TLR5 (PDB: 3J0A), and TLR1-TLR2 (PDB: 2Z7X) complexes using ClusPro 2.0^71^ and HDOCK servers (https://hdock.phys.hust.edu.cn/) as used above.^91^ These innate immune receptors are crucial in recognizing a variety of pathogen-associated molecular patterns (PAMPs), including those present in vaccines and adjuvants. They play a vital role in triggering effective immune responses and ensuring successful vaccine-induced immunity.^92^

To identify the amino acid residues involved in polar contacts and electrostatic clashes within VEC-TLRs complexes, crucial for protein–protein interaction stability, we used PyMol (https://pymol.org/), a widely used software for visualizing and analyzing molecular structures in 3D with a 3.6 Å threshold.^93^

### 3.7 Molecular dynamic simulation

The docked TLR1-TLR2, TLR4, and TLR5 with vaccine complexes were analyzed through molecular dynamics simulations using the online iMODS server (https://imods.chaconlab.org/)^94^ with its default parameters. The iMODS analysis provides key insights into the structural dynamics of the complex, predicting the collective motions of proteins using normal mode analysis (NMA) at internal coordinates. This tool calculates the deformability, eigenvalues, variance, covariance map, B-factor, and elastic network of the vaccine receptor complex.^95^

### 3.8 Prediction of secondary structure and surface accessibility

The secondary and tertiary structures, as well as the solvent accessibility, of the vaccine constructs were analyzed using the NetSurfP-3.0 server (https://services.healthtech.dtu.dk/services/NetSurfP-3.0/).^96^ The neural network NetChop 3.1 was used to analyze peptide cleavage in MHC class-I epitopes (https://services.healthtech.dtu.dk/services/NetChop-3.1/).^97^ Furthermore, the ProsperousPlus server was utilized to predict cathepsin-specific peptidase activity, further validating the processing of these epitopes for MHC presentation (https://prosperous.erc.monash.edu/).^98^ Predicting proteasomal cleavage sites is crucial for identifying potential T-cell epitopes as well as for producing correctly cleaved C-terminal peptides that can trigger robust immune responses.^99^

### 3.9 Discontinuous B-cell epitopes

The selected vaccine constructs were evaluated using the ElliPro server (https://tools.iedb.org/ellipro/) to identify and depict potential antibody-binding regions within the provided protein sequence or structure.^100^ Conformational B-cell epitope analysis was conducted with a minimum score threshold of 0.70 while retaining the default maximum distance setting.

### 3.10 Assessing the immune System's response to the most promising vaccine candidate

The C-ImmSim server was used to model the immune system response to the vaccine (https://150.146.2.1/C-IMMSIM/index.php?page=1).^101^ It employs a position-specific scoring matrix approach for the prediction of immune epitopes and applies machine learning methodologies to model immune interactions across three anatomical regions within mammalian biological systems.^102^ The vaccine treatments utilized the default parameters (Random seed: 12345; Simulation volume: 10), with three vaccine injections administered at 30-day intervals. The time steps for these three injections were set at 1, 90, and 180 days, with the total simulation steps set at 600.^103^

### 3.11 Vaccine’s safety analysis

Sequence similarity between the vaccine construct and host proteins can potentially result in cross-reactivity and autoimmunity.^31^ Therefore, to ensure the immunological safety of the vaccine, the sequence was subjected to protein BLAST (pBLAST) analysis against the Homo sapiens proteome (GCA_000001405.29).

### 3.12 Codon adaptation and *in silico* cloning

The Benchling webserver (https://www.benchling.com/) was employed to perform reverse translation on the sequence. The codon usage bias and GC content of *E. coli* (K12) were considered to optimize the sequence, ensuring the GC content fell within the ideal range of 30–70 %.^104^ Furthermore, the codon adaptation index (CAI) was calculated using the CAI calculator webserver (https://ppuigbo.me/programs/CAIcal/)^105^ and targeted to be greater than 0.8, which is regarded as optimal for achieving high protein expression levels.^106^

The optimized vaccine construct was then engineered using the pET-28a(+) vector, incorporating XhoI and NdeI restriction sites at the C and N termini, respectively. The construct was cloned in-frame with the plasmid's His-tag and thrombin cleavage sites. In addition, *in silico* cloning was conducted utilizing the Benchling webserver (https://www.benchling.com/).

## 4 Availability of data and materials

The data generated or analyzed as part of this study are included in this article, and the experimental datasets are listed below:

(1) NCBI: https://www.ncbi.nlm.nih.gov/

(2) Database of Essential Genes: https://tubic.org/deg/public/index.php

(3) Virulence Factor Database: https://www.mgc.ac.cn/VFs/main.htm

(4) Comprehensive Antibiotic Resistance Database: https://card.mcmaster.ca/

(5)Antibiotic Resistance Gene-ANNOTation database: https://www.mediterranee-infection.com/acces-ressources/base-de-donnees/arg-annot-2/

(6) PSORTb v3.0.2: https://www.psort.org/psortb/

(7) BUSCA server: https://busca.biocomp.unibo.it

(8) VaxiJen v2.0 tool: https://www.ddg-pharmfac.net/vaxijen/VaxiJen/VaxiJen.html

(9) Expasy ProtParam server: https://web.expasy.org/protparam/

(10) DeepTMHMM server: https://dtu.biolib.com/DeepTMHMM

(11) SignalP 6.0: https://services.healthtech.dtu.dk/services/SignalP-6.0/

(12) IEDB Tepitool: https://tools.iedb.org/tepitool/

(13) NetCTLpan 1.1 web server: https://services.healthtech.dtu.dk/service.php?NetCTLpan-1.1

(14) ABCpred servers: https://webs.iiitd.edu.in/raghava/abcpred/

(15) IEDB server: https://tools.immuneepitope.org/immunogenicity/

(16) ToxinPred server: https://www.imtech.res.in/raghava/toxinpred/index.html/

(17) AllerTop server: https://www.ddg-pharmfac.net/AllerTOP/feedback.py

(18) IFNepitope2 server: https://webs.iiitd.edu.in/raghava/ifnepitope2/

(19) PEP-FOLD3 server: https://bioserv.rpbs.univ-paris-diderot.fr/services/PEP-FOLD3/

(20) ClusPro 2.0 server: https://cluspro.bu.edu/login.php?redir=/home.php

(21) HawkDock server: https://cadd.zju.edu.cn/hawkdock/

(22) PRODIGY: https://rascar.science.uu.nl/prodigy/

(23) IEDB population coverage analysis tool: https://tools.iedb.org/conservancy

(24) SOLpro Scratch Protein Predictor: https://scratch.proteomics.ics.uci.edu/

(25) I-TASSER server: https://zhanggroup.org/I-TASSER/

(26) Galaxy Refine web server: https://galaxy.seoklab.org/refine

(27) SAVES v6.1: https://saves.mbi.ucla.edu/

(28) ProSA web server: https://prosa.services.came.sbg.ac.at/prosa.php

(29) HDOCK servers: https://hdock.phys.hust.edu.cn/

(30) Pymol: https://pymol.org/

(31) iMODS server: https://imods.chaconlab.org/

(32) NetSurfP-3.0 server: https://services.healthtech.dtu.dk/services/NetSurfP-3.0/

(33) NetChop 3.1: https://services.healthtech.dtu.dk/services/NetChop-3.1/

(34) ProsperousPlus: https://prosperous.erc.monash.edu/

(35) ElliPro server: https://tools.iedb.org/ellipro/

(36) C-ImmSim server: https://150.146.2.1/C-IMMSIM/index.php?page=1

(37) Benchling webserver: https://www.benchling.com/

## 5 RESULTS

### 5.1 Proteomic analysis of *E. cloacae* core proteins

BPGA version 1.3 identified 2,593 non-paralogous protein sequences from the complete proteomes of 21 *Enterobacter cloacae* samples associated with human infections, including the reference proteome of the *E. cloacae* strain (retrieved from the National Center for Biotechnology Information database https://www.ncbi.nlm.nih.gov/, Table S1) (Table 1).

**Table 1:** Subtractive analysis of the *Enterobacter cloacae* proteome.

Serial No.	Protein Prediction	*Enterobacter cloacae (*No. of proteins)
1	Total overall quantity of proteins from *E. cloacae* reference strain	4.664
2	Proteins that are non-paralogous and part of the core proteome	2.593
3	Essential proteins determined through BLASTp analysis against DEG database (evalue ≤ 10^−4^ ,bitscore > 100, ppos > 60 %)	1.287
4	Resistance factors determined through BLASTp analysis against CARD and ARG-ANNOT database (evalue ≤ 10 − 4, bitscore > 100, ppos > 60 %)	61
5	Virulence factors determined through BLASTp analysis against VFDB database (evalue ≤ 10 − 4, bitscore > 100, ppos > 60 %)	172
6	Non-redundant proteins associated with essentiality, resistance, or virulence	1.352
7	Non-gut homologous proteins (BLASTp, evalue ≤ 10 − 4, ppos > 60 %)	40
8	Non-human and non-gut homologous proteins, (BLASTp, evalue ≤ 10 − 4, ppos > 60 %)	39
9	Cytoplasmatic or Plasmatic Membrane proteins identified using PSORTb and BUSCA servers	27
10	Extracellular or Outer Membrane proteins identified using PSORTb and BUSCA servers	12
11	Surface-exposed proteins exhibiting antigenic properties as determined by VaxiJen v2.0	9
	Selected proteins for epitope screening	9

The Database of Essential Genes showed that 1,287 out of these 2,593 non-paralogous core proteins were found to be essential. Furthermore, 172 were found to be putative virulence factors using the Virulence Factor Database, and 61 were associated with antibiotic resistance according to the Comprehensive Antibiotic Resistance and Antibiotic Resistance Gene-ANNOTation Databases.

A total of 1,352 non-redundant essential, resistance, and/or virulence proteins were identified. These proteins were further screened against 79 gut microbiome proteomes, resulting in the identification of 40 proteins with no similarity to the gut microbiome. Screening against the *Homo sapiens* reference proteome revealed that 39 of these proteins were also non-human homologs. Only 12 out of the 39 proteins were found to be extracellular or in the outer membrane and were taken forward for further analysis (Table 1).

### 5.2 Analysis of transmembrane regions, signal peptides, physicochemical properties, and antigenicity

Analysis of the 12 surface-exposed proteins by SignalP 6.0 revealed that 9 proteins contained secretory signal peptides, transported by the Sec translocon and cleaved by Signal Peptidase I. The remaining 3 proteins contained lipoprotein signal peptides, also transported by the Sec translocon but cleaved by Signal Peptidase II (Sec/SPII). Notably, none of the analyzed proteins had transmembrane helices. Furthermore, all 12 proteins exhibited a negative Grand Average of Hydropathicity (GRAVY) value, indicating solubility and suitability for vaccine construction, as determined by the VaxiJen v2.0 tool. The antigenicity and localization evaluation revealed that 2 of the 12 proteins were identified as outer membrane proteins, specifically multi-drug resistance outer membrane protein MdtQ and Oligogalacturonate-specific porin KdgM. An additional 7 proteins were found to be extracellular proteins with notable antigenic profiles, including Curli major subunit CsgA, putative fimbrial assembly protein SfmF, Curli production assembly/transport protein CsgF, Type 1 fimbria D-mannose-specific adhesin FimH, Curli minor subunit CsgB, Heat shock protein HslJ, and Curli assembly chaperone CsgC. These nine proteins were subsequently evaluated for useful epitopes for vaccine construction. Detailed physicochemical and antigenic properties of these proteins are presented in Table 2.

**Table 2:** Analysis of the function, antigenicity, hydrophobicity, signal peptide, transmembrane helices, and physicochemical properties of target proteins.

RefSeq ID^1^	Protein name^2^	Function^3^	Location^4^	Antigenicity Score^5^	Hydrophobicity (GRAVY)^6^	Length (aa)^7^	Mol. Wt kDa^8^	TMHs^9^	Signal P^3^
WP_023620194.1	*Curli major subunit CsgA [*Enterobacter*]	The primary subunit protein that constitutes the curli fibers, a crucial component of bacterial biofilms.	Extracellular	1.0214	−0.318	153	15.37	0	Signal Peptide (Sec/SPI)
WP_013095935.1	* Putative fimbrial assembly protein SfmF [*Enterobacter*]	Cell adhesion that promotes single-species biofilm formation	Extracellular	0.7398	−0.152	174	18.28	0	Signal Peptide (Sec/SPI)
WP_013097173.1	*Curli production assembly/transport protein CsgF [*Enterobacter*]	Biogenesis of curli organelles	Extracellular	0.5523	−0.315	137	14.98	0	Signal Peptide (Sec/SPI)
WP_013095931.1	Type 1 fimbrial protein subunit FimI [*Enterobacter*]	Cell adhesion that enables the formation of single-species biofilms	Extracellular	0.4642	0.125	181	19.64	0	Signal Peptide (Sec/SPI)
WP_028027948.1	Curli production assembly/transport protein CsgG [*Enterobacter*]	Biogenesis of curli organelles	Extracellular	0.3337	−0.018	277	30.37	0	Lipoprotein signal peptide (Sec/SPII)
WP_182059907.1	*Type 1 fimbria D-mannose specific adhesin FimH [*Enterobacter* sp. RHBSTW-00175]	Cell adhesion involved in single-species biofilm formation	Extracellular	0.6488	−0.161	355	36.35	0	Signal Peptide (Sec/SPI)
WP_182060423.1	*Curli minor subunit CsgB [*Enterobacter* sp. RHBSTW-00175]	Cell adhesion	Extracellular	0.6595	−0.223	151	16.05	0	Signal Peptide (Sec/SPI)
WP_315685900.1	*Heat shock protein HslJ [*Enterobacter* cloacae]	Defense against oxidative stress	Extracellular	0.675	−0.175	146	15.80	0	Lipoprotein signal peptide (Sec/SPII)
WP_166716973.1	*Curli assembly chaperone CsgC [unclassified *Enterobacter*]	Extracellular assembly of CsgA into thin aggregative fimbriae (Tafi) fibers.	Extracellular	0.7714	−0.052	110	11.79	0	Signal Peptide (Sec/SPI)
WP_013097172.1	Curli production assembly/transport protein CsgE [*Enterobacter cloacae*]	Prevents the aggregation of CsgA into Curli fibers	Extracellular	0.3228	−0.064	129	14.40	0	Signal Peptide (Sec/SPI)
WP_226834852.1	*Multi-drug resistance outer membrane protein MdtQ [*Enterobacter cloacae*]	Could be involved in resistance to puromycin, acriflavine and tetraphenylarsonium chloride	Outer Membrane	0.5923	−0.158	472	50.99	0	Lipoprotein signal peptide (Sec/SPII)
WP_020687115.1	*Oligogalacturonate-specific porin KdgM family protein [*Enterobacter*]	Facilitates the transport of oligogalacturonate	Outer Membrane	0.8742	−0.685	227	25.90	0	Signal Peptide (Sec/SPI)

All data were analyzed using various servers: 1, 2 = NCBI; 3 = UNIPROT; 4 = BUSCA and PsortB; 5, 6, 7, 8 = ProtParam; 9 = TMHMM server; 10 = SignalP 6.0. TMHs: transmembrane helices. * Proteins selected for epitopes prediction.

### 5.3 MHC class-I epitopes prediction

Analysis of the selected proteins using Tepitool and NetCTLpan 1.1 identified 144 potential 9 residue T-cell MHC class I epitopes, of which 70 were suggested to be immunogenic by the IEDB. Further analysis revealed that 42 out of the 70 epitopes were antigenic, as determined by the VaxiJen v2.0 server using a threshold of ≥0.5. Importantly, none of these antigenic epitopes were found to be toxic, as assessed using the ToxinPred server, ensuring they could be safely included in any construct. Additionally, their GRAVY values were calculated using the ProtParam server to evaluate the hydrophilic nature of the epitopes. Among the 42 antigenic epitopes, 20 displayed negative GRAVY values, indicating hydrophilicity and potential solubility under physiological conditions.

We further refined these 20 hydrophilic epitopes by analyzing for allergenicity using the AllerTop server. This identified 12 non-allergenic epitopes, and so these were deemed optimal as they met all the criteria for immunogenicity, antigenicity, non-toxicity, non-allergenicity, and negative GRAVY values (Table 3). Consequently, these 12 epitopes continued to molecular docking analysis to explore their interactions with MHC molecules.

**Table 3:** Characteristics of predicted MHC-I epitopes, such as length, sequence, HLA allele coverage, immunogenicity, antigenicity, toxicity, hydrophobicity, and allergenicity.

Target Protein	Length (aa)^1^	Epitope Sequence^2^	HLA Allele coverage^3^	Immunogenicity Score^4^	Antigenicity Score ^5)^	Toxicity^6^	Hydrophobicity (GRAVY)^7^	Allergenicity Potential^8^
Curli minor subunit CsgB	9	GSDLANTEY	HLA-A*01:01 HLA-A*30:02	0.12	0.58	Non-toxin	−0.9	Non-allergen
Putative Fimbrial assembly protein SfmF	9	NPRPGRADA	HLA-B*07:02	0.13	1.91	Non-toxin	−1.78	Non-allergen
Heat shock protein HslJ	9	KLAEGELKV	HLA-A*02:03 HLA-A*02:01 HLA-A*02:06 HLA-A*32:01	0.11	1.28	Non-toxin	−0.18	Non-allergen
	9	QLQNHRFIL	HLA-B*08:01	0.21	0.52	Non-toxin	−0.37	Non-allergen
Multi-drug resistance outer membrane protein MdtQ	9	ELLARRPDL	HLA-B*08:01	0.14	1.22	Non-toxin	−0.49	Non-allergen
	9	SMSEVDAAR	HLA-A*33:01 HLA-A*68:01 HLA-A*31:01	0.16	0.6	Non-toxin	−0.38	Non-allergen
	9	SVARLYWDW	HLA-B*57:01 HLA-A*32:01 HLA-B*58:01 HLA-B*53:01	0.25	1.56	Non-toxin	−0.23	Non-allergen
Oligogalacturonate-specific porin KdgM family protein	9	ESNDSRTIY	HLA-A*26:01 HLA-A*01:01 HLA-B*35:01 HLA-A*30:02 HLA-B*15:01 HLA-B*53:01	0.02	1.34	Non-toxin	−1.57	Non-allergen
	9	HEDQISWRW	HLA-B*44:02 HLA-B*44:03 HLA-B*53:01 HLA-B*58:01 HLA-B*40:01 HLA-B*57:01	0.08	2.24	Non-toxin	−1.81	Non-allergen
	9	VAARYRYEY	HLA-A*30:02 HLA-B*35:01 HLA-B*58:01 HLA-B*57:01 HLA-A*01:01 HLA-A*32:01 HLA-B*15:01 HLA-B*53:01	0.17	1.19	Non-toxin	−0.96	Non-allergen
Type1 fimbria D-mannose specific adhesin FimH	9	DSKLVFRLR	HLA-A*33:01 HLA-A*68:01 HLA-A*31:01	0.11	1.17	Non-toxin	−0.29	Non-allergen
	9	ITTEIDSYK	HLAA*11:01 HLA-A*68:01 HLA-A*03:01	0.12	0.84	Non-toxin	−0.6	Non-allergen

All data were analyzed using various servers: 1 = ProtParam; 2, 3 = Tepitool and NetCTLpan1.1; 4 = IEDB server; 5 = VaxiJen v2.0; 6 = ToxinPred; 7 = ProtParam; 8 = AllerTop.

### 5.4 MHC Class-II epitopes prediction

The analysis of MHC class II epitopes from 9 proteins (Table 2) identified 319 high-affinity, 15-residue epitopes using the Tepitool server. Of these identified epitopes, 170 were determined to be antigenic using the VaxiJen v2.0 server with a threshold of ≥0.5. One hundred and thirty-five were then found to exhibit negative GRAVY values with the ProtParam server, and so suitable for our purposes. Subsequent toxicity assessment using the ToxinPred server revealed that 131 of these hydrophilic epitopes were non-toxic, confirming their safety for further analysis.

The allergenicity of the 131 non-toxic epitopes was again assessed using the AllerTop server, with 47 epitopes identified as non-allergenic and thus satisfied crucial safety and compatibility criteria. These 47 epitopes were then analyzed for their ability to induce interferon-gamma (IFN-γ), a key cytokine for modulating immune responses. Using the IFNepitope2 server, 9 epitopes were found to have an IFN-γ-inducing profile (Table 4). These 9 epitopes were selected for advanced molecular docking analysis to evaluate their potential interactions with MHC class II molecules.

**Table 4:** Characteristics of MHC-II epitopes, including analysis of their length, epitope sequence, HLA allele coverage, antigenicity, hydrophobicity, toxicity, allergenicity, and ability to induce IFN-γ production.

Target Protein	Length (aa)^1^	Epitope Sequence^2^	HLA Allele coverage^3^	Antigenicity Score^4^	Hydrophobicity (GRAVY)^5^	Toxicity^6^	Allergenicity^7^	IFN-γ inducing Potential^8^
Curli assembly chaperone CsgC	15	LSSQITFKTSQQGNM	HLA-DQA1*04:01 DQB1*04:02	0.83	−0.61	Non-toxin	Non-allergen	Positive
	15	SSQITFKTSQQGNMT	HLA-DRB1*08:02	0.95	−0.91	Non-toxin	Non-allergen	Positive
Curli minor subunit CsgB	15	SQGGYGNTAKIIQQG	HLA-DRB1*11:01	0.84	−0.77	Non-toxin	Non-allergen	Positive
Curli production assembly/transport protein CsgF	15	AFMLNEAQAQNSYKD	HLA-DQA1*01:02 DQB1*06:02 HLA-DQA1*05:01 DQB1*03:01	0.61	−0.87	Non-toxin	Non-allergen	Positive
	15	NEAQAQNSYKDPSFK	HLA-DQA1*01:01 DQB1*05:01	1.06	−1.79	Non-toxin	Non-allergen	Positive
	15	QLQLNVTDRKTGKTS	HLA-DRB1*11:01	1.84	−1.19	Non-toxin	Non-allergen	Positive
Heat shock protein HslJ	15	AQSDISLTKNMTVSG	HLA-DRB1*07:01	0.83	−0.16	Non-toxin	Non-allergen	Positive
Oligogalacturonate-specific porin KdgM family protein	15	KLAYKWKNWAPYVE	HLA-DRB3*02:02	0.53	−1.11	Non-toxin	Non-allergen	Positive
Type 1 fimbria D-mannose specific adhesin FimH	15	QVGIRAWPVSITGNK	HLA-DQA1*01:01 DQB1*05:01 HLA-DQA1*04:01 DQB1*04:02 HLA-DRB1*15:01	1.15	−0.07	Non-toxin	Non-allergen	Positive

All data were analyzed using various servers: 1 = ProtParam; 2, 3 = Tepitool; 4 = VaxiJen v2.0; 5 = ProtParam; 6 = ToxinPred; 7 = AllerTop; 8 = IFNepitope2.

### 5.5 Peptide modeling and molecular docking analysis

Molecular docking analysis was used to identify epitopes with a high promiscuity in binding to multiple HLA alleles, ensuring broad population coverage.^35^, ^107^ The predicted structures were docked with 9 MHC class I alleles and 7 MHC class II alleles using the ClusPro 2.0 and Hawkdock servers to evaluate binding interactions. The epitopes with the lowest MM/GBSA (−44.81 for MHC-I and −46.37 for MHC-II) and binding affinity values (−11.89 for MHC-I and −13.65 for MHC-II), indicating the strongest binding interactions, were selected for formulating the vaccine candidates (Table 5, Table 6).

**Table 5:** Molecular docking analysis between HLA alleles and MHC-I epitopes, assessing the corresponding binding free energy and binding affinity.

Epitopes /Alleles	HLA-A0101 (PDB: 6AT9)		HLA-A0201 (PDB: 3UTQ)		HLA-B1501 (PDB: 1XR8)		HLA-B3501 (PDB: 1ZSD)		HLA-B3901 (PDB: 4O2E)		HLA-B5301 (PDB: 1A1M)		HLA-B5801 (PDB: 5IM7)		HLA-B4403 (PDB: 1SYS)		HLA-A2.1 (PDB: 1I1Y)		Average	
	MM/GBSA	Binding affinity kcal/mol	MM/GBSA	Binding affinity kcal/mol	MM/GBSA	Binding affinity kcal/mol	MM/GBSA	Binding affinity kcal/moll	MM/GBSA	Binding affinity kcal/mol	MM/GBSA	Binding affinity kcal/mol	MM/GBSA	Binding affinity kcal/mol	MM/GBSA	Binding affinity kcal/mol	MM/GBSA	Binding affinity kcal/mol	MM/GBSA	Binding affinity kcal/mol
*DSKLVFRLR	−45.81	−11.00	−39.91	−11.80	−49.78	−9.60	−41.32	−10.70	−46.70	−11.80	−31.34	−10.60	−51.54	−11.40	−53.49	−12.90	−43.36	−11.20	−44.81	−11.22
ELLARRPDL	−26.29	−10.70	−25.85	−11.10	−37.04	−8.60	−30.36	−9.60	−22.95	−12.00	−25.31	−10.30	−31.75	−10.80	−26.45	−11.60	−41.85	−10.40	−29.76	−10.57
*ESNDSRTIY	−40.00	−11.70	−36.87	−11.80	−30.15	−10.90	−25.08	−10.80	−33.62	−11.80	−38.09	−10.50	−31.02	−11.70	−40.97	−12.40	−34.16	−11.20	−34.44	−11.42
*GSDLANTEY	−31.64	−8.10	−42.45	−9.90	−28.09	−7.60	−50.15	−7.70	−24.41	−10.40	−48.11	−7.70	−34.57	−9.70	−23.50	−11.50	−20.90	−9.80	−33.76	−9.16
*HEDQISWRW	−37.50	−11.70	−44.20	−11.90	−36.17	−9.40	−31.07	−10.30	−28.18	−13.30	−26.85	−12.10	−31.48	−10.70	−39.98	−11.40	−30.96	−9.00	−34.04	−11.09
ITTEIDSYK	−22.70	−9.90	−15.16	−9.00	−25.66	−10.90	−28.73	−9.90	−26.45	−12.30	−22.85	−10.30	−25.69	−9.60	−32.18	−10.50	−24.22	−9.00	−24.85	−10.16
KLAEGELKV	−23.38	−9.60	−34.16	−9.10	−20.18	−10.90	−45.33	−9.10	−23.21	−10.10	−39.06	−10.70	−32.15	−9.80	−22.96	−10.70	−27.58	−9.40	−29.78	−9.93
*NPRPGRADA	−33.84	−10.40	−30.81	−12.00	−33.75	−9.90	−34.83	−10.30	−35.69	−10.40	−41.44	−10.50	−39.14	−11.20	−43.20	−11.80	−36.00	−9.50	−36.52	−10.67
*QLQNHRFIL	−33.08	−10.50	−23.87	−12.80	−27.49	−13.10	−28.72	−9.40	−32.42	−12.20	−41.10	−11.20	−38.17	−11.10	−33.48	−12.40	−34.70	−14.30	−32.56	−11.89
SMSEVDAAR	−31.91	−8.90	−31.47	−7.90	−26.68	−12.00	−26.23	−11.20	−36.22	−8.90	−32.41	−12.10	−40.78	−12.20	−22.13	−10.50	−23.85	−12.30	−30.19	−10.67
SVARLYWDW	−26.20	−10.60	−34.49	−9.90	−24.10	−8.30	−27.49	−8.20	−32.90	−11.20	−32.77	−9.20	−33.92	−11.20	−30.53	−9.50	−33.59	−8.20	−30.67	−9.59
*VAARYRYEY	−37.02	−11.00	−42.49	−11.80	−39.46	−9.60	−43.33	−10.70	−45.37	−11.80	−39.15	−11.10	−32.78	−11.40	−31.89	−10.20	−29.38	−11.60	−37.87	−7.84

*Epitopes selected for vaccine construction. The PEP-FOLD3 server was used to generate 3D structures. The Hawkdock server was employed for docking and provided MM/GBSA scores, while Binding affinity scores were obtained from Prodigy. MM/GBSA: molecular mechanics energies combined with the generalized Born and surface area continuum solvation.

**Table 6:** Molecular docking analysis between HLA alleles and MHC-II epitopes and evaluating the corresponding binding free energy and binding affinity.

Epitopes / Alleles	HLA-DRB10101 (PDB: 2FSE)		HLA-DRB10301 (PDB: 1A6A)		HLA-DRB10401 (PDB: 2SEB)		HLA-DRB11501 (PDB: 1BX2)		HLA-DRB30101 (PDB: 2Q6W)		HLA-DRB30202 (PDB: 3C5J)		HLA-DRB5*0101 (PDB: 1H15)		Average	
	MM/GBSA	Binding affinity kcal/mol	MM/GBSA	Binding affinity kcal/mol	MM/GBSA	Binding affinity kcal/mol	MM/GBSA	Binding affinity kcal/mol	MM/GBSA	Binding affinity kcal/mol	MM/GBSA	Binding affinity kcal/mol	MM/GBSA	Binding affinity kcal/mol	MM/GBSA	Binding affinity kcal/mol
AFMLNEAQAQNSYKD	−37.36	−13.10	−22.41	−9.40	−15.45	−11.60	−40.82	−12.20	−39.90	−13.20	−30.17	−11.20	−30.16	−13.20	−30.90	−11.80
*AQSDISLTKNMTVSG	−25.99	−13.10	−35.83	−12.60	−33.82	−12.40	−34.22	−10.60	−26.79	−13.20	−48.11	−12.30	−39.78	−14.30	−34.93	−12.57
*KLAYKWDKNWAPYVE	−40.38	−10.10	−30.81	−10.00	−42.40	−9.60	−39.66	−12.10	−38.94	−13.80	−36.48	−11.80	−45.60	−11.90	−39.18	−11.53
*LSSQITFKTSQQGNM	−36.07	−12.10	−32.65	−13.30	−51.95	−13.30	−44.14	−12.40	−38.69	−13.60	−48.70	−12.70	−44.21	−16.60	−42.34	−13.65
NEAQAQNSYKDPSFK	−27.00	−11.80	−25.94	−9.40	−35.41	−9.00	−26.76	−10.10	−30.63	−11.30	−30.21	−9.20	−35.02	−14.10	−30.14	−10.52
*QLQLNVTDRKTGKTS	−46.39	−13.50	−38.54	−11.50	−38.12	−12.50	−46.82	−11.60	−48.35	−13.10	−45.49	−12.60	−47.58	−14.20	−44.47	−12.58
*QVGIRAWPVSITGNK	−44.93	−14.10	−40.89	−10.30	−43.56	−13.60	−46.45	−12.40	−38.14	−14.70	−47.37	−12.90	−63.27	−15.60	−46.37	−13.25
*SQGGYGNTAKIIQQG	−40.31	−12.80	−34.73	−10.20	−38.51	−10.40	−34.52	−11.80	−37.60	−12.40	−39.54	−10.70	−34.89	−13.10	−37.16	−11.43
*SSQITFKTSQQGNMT	−31.70	−12.50	−33.67	−12.90	−39.93	−13.20	−38.07	−11.60	−34.38	−13.10	−49.37	−11.80	−55.00	−14.80	−40.30	−12.90

* Epitopes selected for vaccine construction. 3D structures were generated using the PEP-FOLD3 server. Molecular docking was carried out using the Hawkdock server. MM/GBSA and binding affinity scores were obtained from Hawkdock and Prodigy, respectively. MM/GBSA: molecular mechanics energies combined with the generalized Born and surface area continuum solvation.

### 5.6 B-cell epitope prediction

In total, 26 linear B-cell epitopes, ranging from 11 to 18 amino acids in length, were identified across 9 proteins (Table 2). Of these, 15 epitopes were found to be both antigenic and non-toxic. Further analysis showed that 10 of these epitopes were also non-allergenic and had a negative GRAVY score. These 10 epitopes, derived from 7 different proteins, were selected for additional studies (Table 7). The linear B cell epitopes predicted using the IEDB Kolaskar and Tongaonkar antigenicity scale are shown in Fig. S1, with higher scoring residues highlighted in yellow.

**Table 7:** Predicted linear B-cell epitopes with their antigenic and toxicity, allergenic, and hydrophobicity properties.

Target Protein	Epitope Sequence^1^	Length (aa)^2^	Antigenicity Score^3^	Toxicity^4^	Allergenicity Risk^5^	Hydrophobicity (GRAVY)^6^
Heat shock protein HslJ	PGKASVQADQLQNH	14	0.8880	Non-toxin	Non-allergen	−1.13
Multi-drug resistance outer membrane protein MdtQ	EVDAARAAFYP	11	0.7752	Non-toxin	Non-allergen	−0.02
	LTAKNQHQQQVEKDAAR	17	1.1507	Non-toxin	Non-allergen	−1.61
Putative Fimbrial assembly protein SfmF	KTDGSDSGLLALNDASTA	18	0.8524	Non-toxin	Non-allergen	−0.32
Curli major subunit CsgA	HGHGQGGNGPNSTLNI	16	2.7205	Non-toxin	Non-allergen	−1.08
Curli production assembly/transport protein CsgF	TGKPGRMVTNDFIVDI	16	0.7441	Non-toxin	Non-allergen	−0.04
Type 1 fimbria D-mannose specific adhesin FimH	ATESGTPLTPNNLTSK	16	0.6860	Non-toxin	Non-allergen	−0.81
	QVGIRAWPVSITGNKP	16	1.1205	Non-toxin	Non-allergen	−0.16
	PKGTSGKSTMRSYVTD	16	1.2373	Non-toxin	Non-allergen	−1.12
Curli minor subunit CsgB	SVVSQDGVGNRARVDQ	16	1.6157	Non-toxin	Non-allergen	−0.64

All data were analyzed using various servers: 1 = IEDB and ABCpred servers; 2 = ProtParam; 3 = VaxiJen v2.0; 4 = ToxinPred; 5 = AllerTop; 6 = ProtParam.

### 5.7 Multi-epitope vaccine design and physicochemical analysis

The screened epitopes mentioned above were combined to create four vaccine candidates (VEC1-4). These epitopes were assembled using the PADRE sequence with various linkers and adjuvants (see Materials and Methods for exact details). The vaccine constructs were of a consistent size and weight (60.34 to 98.51 kDa) with theoretical isoelectric points between 9.42 and 9.69, indicating they carried a negative charge at pH levels above their isoelectric point and a positive charge below it. In addition, the instability index values of the vaccine constructs ranged from 20.10 to 28.88, indicating that the constructs were stable across a wide range of conditions. Stability was further demonstrated at varying temperatures, with the aliphatic index values gained ranging from 67.09 to 71.88. Furthermore, all four vaccine constructs were found to be non-allergenic, antigenic, and have negative GRAVY values indicating solubility and ability to interact efficiently with the immune system. The solubility was confirmed with the SOLpro server, which showed the lowest solubility score at 0.718520 and the highest at 0.927365, suggesting a high probability of being soluble when expressed by *E. coli* (Table 8).

**Table 8:** Four multi-epitope vaccine constructs against *E. cloacae* and their antigenicity, allergenicity, signal peptide properties, hydrophobicity, solubility, and physicochemical properties.

Vaccine name/adjuvant	Sequence	Antigeniciy^1^	Allergenicity^2^	Length (aa)^3^	Mol. weight kDa^3^	pI^3^	Instability index^3^	Aliphatic index^3^	Hydrophobicity (GRAVY).^3^	Solubility Score^4^
VEC1/(50 s ribosomal L7/L12 protein)	EAAAKMAKLSTDELLDAFKEMTLLELSDFVKKFEETFEVTAAAPVAVAAAGAAPAGAAVEAAEEQSEFDVILEAAGDKKIGVIKVVREIVSGLGLKEAKDLVDGAPKPLLEKVAKEAADEAKAKLEAAGATVTVKEAAAK*AKFVAAWTLKAAA*AAYDSKLVFRLRAAYESNDSRTIYAAYGSDLANTEYAAYHEDQISWRWAAYNPRPGRADAAAYQLQNHRFILAAYVAARYRYEYGPGPGAQSDISLTKNMTVSGGPGPGKLAYKWDKNWAPYVEGPGPGLSSQITFKTSQQGNMGPGPGQLQLNVTDRKTGKTSGPGPGQVGIRAWPVSITGNKGPGPGSQGGYGNTAKIIQQGGPGPGSSQITFKTSQQGNMTKKPGKASVQADQLQNHKKEVDAARAAFYPKKLTAKNQHQQQVEKDAARKKKTDGSDSGLLALNDASTAKKHGHGQGGNGPNSTLNIKKTGKPGRMVTNDFIVDIKKATESGTPLTPNNLTSKKKQVGIRAWPVSITGNKPKKPKGTSGKSTMRSYVTDKKSVVSQDGVGNRARVDQAAY*AKFVAAWTLKAAA*AAY	0.9062	Non-allergen	572	60.34	9.66	20.10	68.76	−0.52	0.883904
VEC2/(HBHA)	EAAAKMAENPNIDDLPAPLLAALGAADLALATVNDLIANLRERAEETRAETRTRVEERRARLTKFQEDLPEQFIELRDKFTTEELRKAAEGYLEAATNRYNELVERGEAALQRLRSQTAFEDASARAEGYVDQAVELTQEALGTVASQTRAVGERAAKLVGIELEAAAK*AKFVAAWTLKAAA*AAYDSKLVFRLRAAYESNDSRTIYAAYGSDLANTEYAAYHEDQISWRWAAYNPRPGRADAAAYQLQNHRFILAAYVAARYRYEYGPGPGAQSDISLTKNMTVSGGPGPGKLAYKWDKNWAPYVEGPGPGLSSQITFKTSQQGNMGPGPGQLQLNVTDRKTGKTSGPGPGQVGIRAWPVSITGNKGPGPGSQGGYGNTAKIIQQGGPGPGSSQITFKTSQQGNMTKKPGKASVQADQLQNHKKEVDAARAAFYPKKLTAKNQHQQQVEKDAARKKKTDGSDSGLLALNDASTAKKHGHGQGGNGPNSTLNIKKTGKPGRMVTNDFIVDIKKATESGTPLTPNNLTSKKKQVGIRAWPVSITGNKPKKPKGTSGKSTMRSYVTDKKSVVSQDGVGNRARVDQAAY*AKFVAAWTLKAAA*AAY	0.9303	Non-allergen	601	64.53	9.69	26.39	67.09	−0.66	0.894815
VEC3/(HBHA Conserved Sequence)	EAAAKMAENSNIDDIKAPLLAALGAADLALATVNELITNLRERAEETRRSRVEESRARLTKLQEDLPEQLTELREKFTAEELRKAAEGYLEAATSELVERGEAALERLRSQQSFEEVSARAEGYVDQAVELTQEALGTVASQVEGRAAKLVGIELEAAAK*AKFVAAWTLKAAA*AAYDSKLVFRLRAAYESNDSRTIYAAYGSDLANTEYAAYHEDQISWRWAAYNPRPGRADAAAYQLQNHRFILAAYVAARYRYEYGPGPGAQSDISLTKNMTVSGGPGPGKLAYKWDKNWAPYVEGPGPGLSSQITFKTSQQGNMGPGPGQLQLNVTDRKTGKTSGPGPGQVGIRAWPVSITGNKGPGPGSQGGYGNTAKIIQQGGPGPGSSQITFKTSQQGNMTKKPGKASVQADQLQNHKKEVDAARAAFYPKKLTAKNQHQQQVEKDAARKKKTDGSDSGLLALNDASTAKKHGHGQGGNGPNSTLNIKKTGKPGRMVTNDFIVDIKKATESGTPLTPNNLTSKKKQVGIRAWPVSITGNKPKKPKGTSGKSTMRSYVTDKKSVVSQDGVGNRARVDQAAY*AKFVAAWTLKAAA*AAY	0.9366	Non-allergen	592	63.41	9.64	28.88	68.58	−0.65	0.927365
VEC4/(FliC flagellin)	EAAAKMAQVINTNSLSLLTQNNLNKSQSALGTAIERLSSGLRINSAKDDAAGQAIANRFTANIKGLTQASRNANDGISIAQTTEGALNEINNNLQRVRELAVQSANSTNSQSDLDSIQAEITQRLNEIDRVSGQTQFNGVKVLAQDNTLTIQVGANDGETIDIDLKQINSQTLGLDTLNVQQKYKVSDTAATVTGYADTTIALDNSTFKASATGLGGTDQKIDGDLKFDDTTGKYYAKVTVTGGTGKDGYYEVSVDKTNGEVTLAGGATSPLTGGLPATATEDVKNVQVANADLTEAKAALTAAGVTGTASVVKMSYTDNNGKTIDGGLAVKVGDDYYSATQNKDGSISINTTKYTADDGTSKTALNKLGGADGKTEVVSIGGKTYAASKAEGHNFKAQPDLAEAAATTTENPLQKIDAALAQVDTLRSDLGAVQNRFNSAITNLGNTVNNLTSARSRIEDSDYATEVSNMSRAQILQQAGTSVLAQANQVPQNVLSLLREAAAK*AKFVAAWTLKAAA*AAYDSKLVFRLRAAYESNDSRTIYAAYGSDLANTEYAAYHEDQISWRWAAYNPRPGRADAAAYQLQNHRFILAAYVAARYRYEYGPGPGAQSDISLTKNMTVSGGPGPGKLAYKWDKNWAPYVEGPGPGLSSQITFKTSQQGNMGPGPGQLQLNVTDRKTGKTSGPGPGQVGIRAWPVSITGNKGPGPGSQGGYGNTAKIIQQGGPGPGSSQITFKTSQQGNMTKKPGKASVQADQLQNHKKEVDAARAAFYPKKLTAKNQHQQQVEKDAARKKKTDGSDSGLLALNDASTAKKHGHGQGGNGPNSTLNIKKTGKPGRMVTNDFIVDIKKATESGTPLTPNNLTSKKKQVGIRAWPVSITGNKPKKPKGTSGKSTMRSYVTDKKSVVSQDGVGNRARVDQAAY*AKFVAAWTLKAAA*AAY	0.9330	Non-allergen	937	98.51	9.42	22.70	71.88	−0.55	0.718520

VEC: *Enterobacter cloacae* vaccine. Sequences in bold represent the linker sequences (EAAAK and AAY). The italic regions represent PADRE sequences (AKFVAAWTLKAAA). 1: VaxiJen v2.0; 2: AllerTOP v2.0; 3: ProtParam Expasy; 4: SOLPro. Isoelectric point (pI).

### 5.8 Peptide cleavage analysis and population coverage evaluation

The potential efficacy of our vaccine candidates was evaluated through proteasomal and cathepsin-specific peptidase activity with peptide cleavage analysis using NetChop3.1 and ProsperousPlus. This analysis revealed 16 proteasomal cleavage sites within the MHC class I epitope clusters, with many of these sites positioned near the selected “AAY” linker. Additionally, we identified numerous cleavage sites for various cathepsins, including ten for cathepsin G, seven for cathepsin K, three for cathepsin E, four for cathepsin D, 12 for cathepsin S, and 12 for cathepsin B. All these cleavage sites had a score greater than 0.8 and were situated within the MHC class II epitope clusters. These findings suggest the multi-epitope vaccine construct is well-positioned for effective processing and presentation via both MHC class I and MHC class II molecules, ensuring a broad immune response (Fig. S2).

**Fig. 3:**
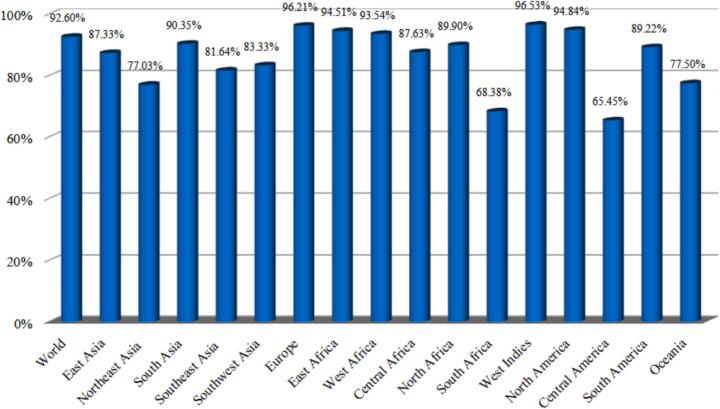
Population coverage analysis of the prioritized MHC I/II epitopes selected for vaccine construction.

MHC molecules are highly polymorphic, with more than 7000 distinct human HLA alleles identified, each exhibiting varying frequencies across diverse ethnic groups and geographic regions.^94^ Therefore, we used the IEDB population coverage tool to ensure our vaccine candidates have the ability to be recognized by individuals from different populations. The analysis revealed that the MHC I and MHC II epitope clusters, included in our vaccine constructs, can cover approximately 92.60 % of the global population. Specifically, the coverage was higher in the West Indies (96.53 %) and Europe (96.21 %), while it was lowest in South Africa (68.38 %) and Central America. (65.45 %) (Fig. 3).

### 5.9 3D structural prediction, refinement, and validation for *e. cloacae* vaccines constructs

**Fig. 4:**
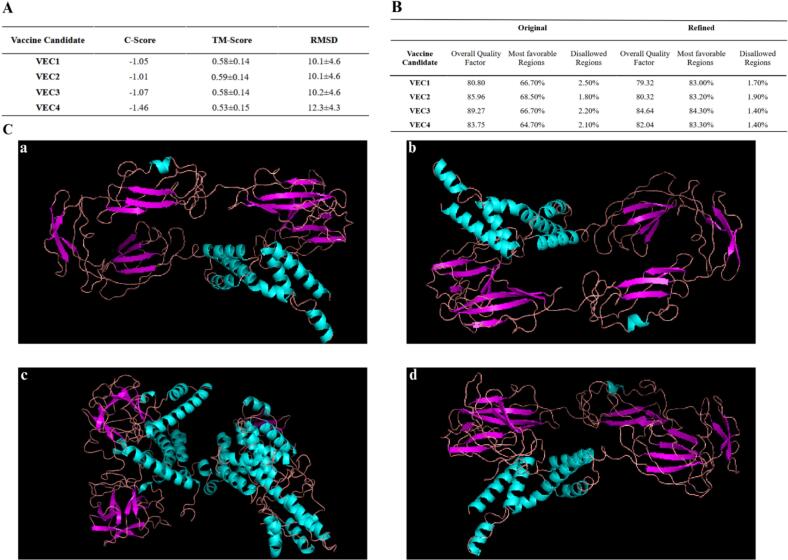
Computational modeling and structural refinement of *E. cloacae* vaccine candidates. (A) Quality assessment of four vaccine constructs (VEC1-4) using I-TASSER-derived metrics (C-Score, TM-Score, and RMSD). (B) GalaxyRefine validation before (left) and after (right) refinement: highlighting the overall quality factor, percentage of residues in the most favorable regions, and disallowed regions. (C) Final 3D structures colored by secondary elements, with helices in cyan, beta sheets in magenta, and loops/coils in light pink. Individual models are as follows: VEC1 (a), VEC2 (b), VEC3 (c), and VEC4 (d).

The 3D structure of each vaccine candidate was determined through threading analysis using the I-TASSER web server. Each sequence generated five predicted 3D models, with the first model being selected for further investigation based on C-score values of −1.05, −1.01, −1.07, and −1.46 for VEC1 to 4, respectively (Fig. 4A). C-scores assess the similarity between the query and template structures, considering the significance of the threading template alignment and query coverage.^43^ Additionally, the structural similarity between our protein models was analyzed using I-TASSER, which computed the root mean square deviation (RMSD) of the atomic positions and provided a template modeling score (TM-score). The TM scores for the vaccine models ranged from 0.53 ± 0.15 to 10.1 ± 0.46, while the RMSD values varied between 0.59 ± 0.14 and 12.3 ± 4.3 (Fig. 4A).

The 3D models derived from each vaccine construct were further optimized by utilizing the GalaxyRefine server. The refinement process produced five models for each vaccine construct, with the first refined model of each construct identified as the most suitable and subsequently selected. These refined models were then assessed for structural integrity through various validation tools, including ERRAT, PROCHECK's Ramachandran plot, and ProSA web servers. ERRAT specifically presents a general structure quality score. Prior to refinement, the scores varied between 89.27 (VEC3) and 80.80 (VEC1), with VEC2 and VEC4 having scores of 85.96 and 83.75, respectively. However, after refinement, the quality scores for VEC1-4 were 79.32, 80.32, 84.64, and 82.03, respectively (Fig. 4B). Ramachandran plot analysis showed significant improvements in the distribution of residues within the allowed regions. In the refined models, 83.00 %, 83.20 %, 84.30 %, and 83.30 % of the residues in VEC1, VEC2, VEC3, and VEC4 were in the allowed regions, all showing improvement over the originals (Fig. 4B). Refinement also reduced the percentage of residues situated in disallowed regions by around half for all constructs (Fig. 4B). The Z-scores predicted by ProSa-Web for the four refined vaccine models VEC1, VEC2, VEC3, and VEC4 were −4.66, −4.52, −4.04, and −4.09, respectively. While the Z-score deviated slightly from the typical range observed for natural proteins of comparable size, the ERRAT analysis yielded a score (79 to 84) over 50, indicating the models are of high quality.^108^ Additionally, the Ramachandran plot analysis demonstrated that more than 80 % of the residues were classified in the favored region, validating the accuracy of the tertiary structure of the vaccines (Fig. S3). 3D models of all vaccines are shown in Fig. 4C.

### 5.10 Molecular docking analysis of vaccine candidates targeting toll-like receptors

Protein-protein docking analysis was performed to evaluate the interaction potential of VEC1, VEC2, VEC3, and VEC4 against the TLR4-human MD2 complex (PDB:3FXI), TLR1-TLR2 heterodimer (PDB:2Z7X), and against TLR5 (PDB:3J0A). VEC1 demonstrated the highest average binding affinity (−24.07 kcal/mol) and docking score (–322.21) with the most favorable dissociation constant (Kd) at 37 °C, making it the most promising vaccine candidate targeting *E. cloacae* (Table 9).

**Table 9:** Comparative analysis of molecular docking scores, confidence scores, binding affinity values, and dissociation constants (Kd) for the binding of vaccine candidates with Toll-like receptors.

Toll-like Receptor	TLR1/2				TLR4				TLR5				Average		
Vaccine Candidate	Docking Score	Confidence Score	Binding Affinity (kcal/mol)	Kd (M) at 37℃	Docking Score	Confidence Score	Binding Affinity (kcal/mol)	Kd (M) at 37℃	Docking Score	Confidence Score	Binding Affinity (kcal/mol)	Kd (M) at 37℃	Docking Score	Binding Affinity (kcal/mol)	Kd (M) at 37℃
VEC1	−311.51	0.96	−25.00	2.5e^-18^	−301.26	0.95	−22.60	1.2e^-16^	−353.87	0.98	−24.60	4.3e^-18^	−322.21	−24.07	4.23e^-17^
VEC2	−320.83	0.97	−24.80	3.5e^-18^	−282.41	0.93	−21.60	6.0e^-16^	−344.44	0.98	−24.50	5.2e^-18^	−315.89	−23.63	2.03e^-16^
VEC3	−283.19	0.93	−23.70	1.8e^-17^	−328.35	0.97	−19.60	1.6e^-14^	−299.81	0.95	−20.60	3.0e^-15^	−303.14	−21.30	6.34e^-15^
VEC4	−276.66	0.93	−17.90	2.3e^-13^	−280.86	0.93	−20.20	5.6e^-15^	−309.49	0.96	−17.70	3.4e^-13^	−289.00	−18.60	1.92e^-13^

**Fig. 5:**
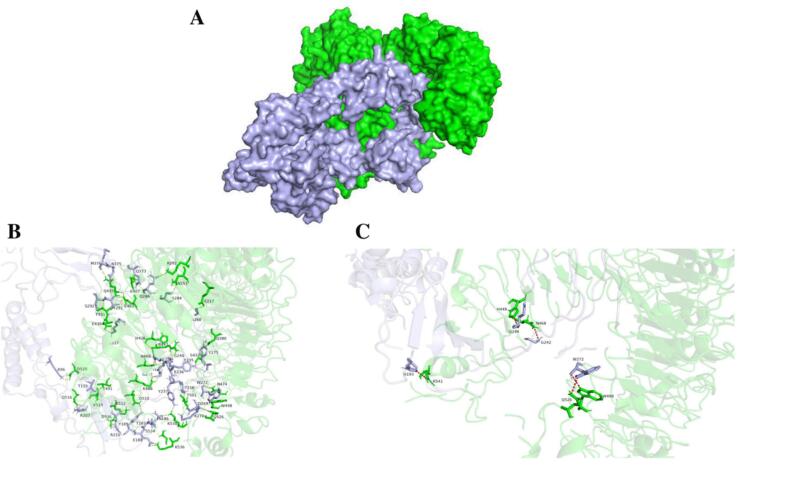
Molecular interactions between vaccine candidate VEC1 and TLR1/2 heterodimer (A) Overall 3D structure of the VEC1-TLR1/2 complex, with VEC1 shown in light blue and TLR1/2 in green. (B) Close-up view of polar contacts at the binding interface, with interacting residues represented as stick models and labeled. (C) Electrostatic interactions between VEC1 and TLR1/2, highlighting charge complementarity, with clashing residues shown as labeled sticks.

**Fig. 6:**
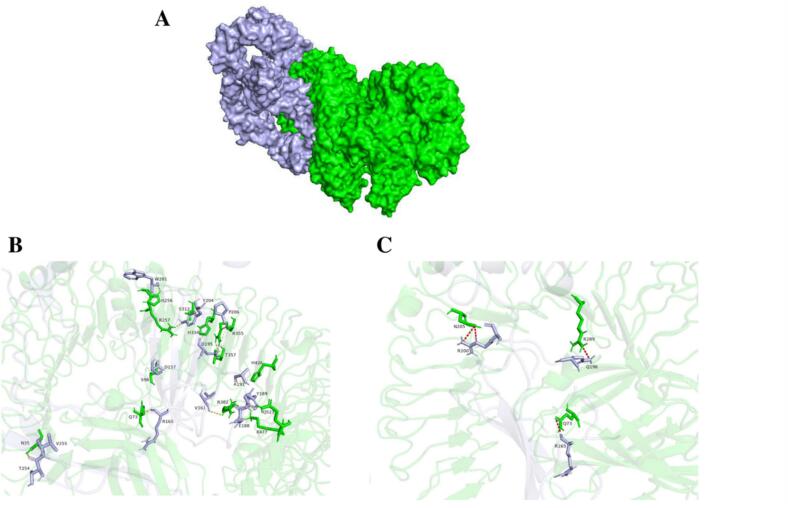
Molecular interactions between vaccine candidate VEC1 and TLR4. (A) Overall 3D structure of the VEC1-TLR4 complex, with VEC1 shown in light blue and TLR4 in green. (B) Close-up view of polar contacts at the binding interface, with interacting residues represented as stick models and labeled. (C) Electrostatic interactions between VEC1 and TLR4, highlighting charge complementarity, with clashing residues shown as labeled sticks.

**Fig. 7:**
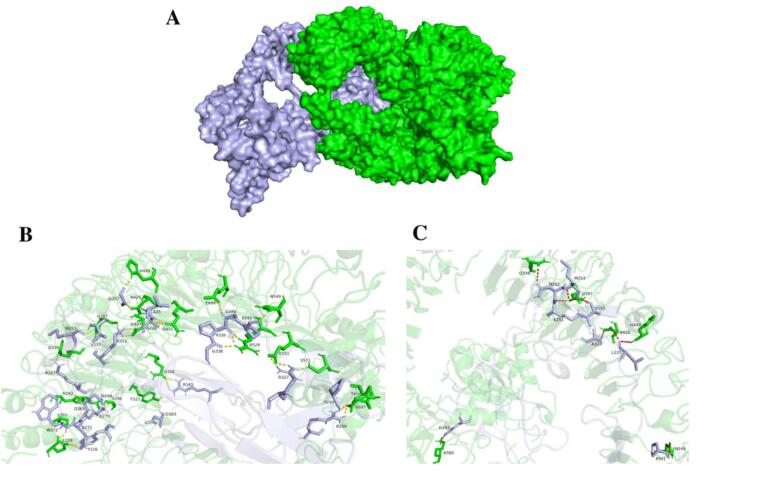
Molecular interactions between vaccine candidate VEC1 and TLR5. (A) Overall 3D structure of the VEC1-TLR5 complex, with VEC1 shown in light blue and TLR5 in green. (B) Close-up view of polar contacts at the binding interface, with interacting residues represented as stick models and labeled. (C) Electrostatic interactions between VEC1 and TLR5, highlighting charge complementarity, with clashing residues shown as labeled sticks.

The surface representations of the VEC1-TLR1/2, VEC1-TLR4, and VEC1-TLR5 complexes are depicted in Figs. 5A, 6A, and 7A, respectively. For each complex, the residue interactions between VEC1 and the TLRs were analyzed by examining the polar contacts (Figs. 5B, 6B, and 7B) and electrostatic clashes (Figs. 5C, 6C, and 7C) using PyMol. The TLRs are shown in green, while VEC1 is depicted in light blue. In all VEC1-TLR complexes, multiple residues are involved in polar interactions and electrostatic clashes. However, no significant differences were observed in the number of electrostatic clashes among the complexes. A total of 15 residues from TLR1/2 chain A and 11 residues from TLR1/2 chain B engage in polar interactions with 26 residues of VEC1 (Fig. 5B). Notably, the VEC1-TLR1/2 complex displayed a higher number of polar interactions compared to the other complexes (Fig.s 5B, 6B, 7B), indicating that hydrogen bonding is a key factor in determining the affinity and stability of the complex.

### 5.11 Molecular dynamics simulations

**Fig. 8:**
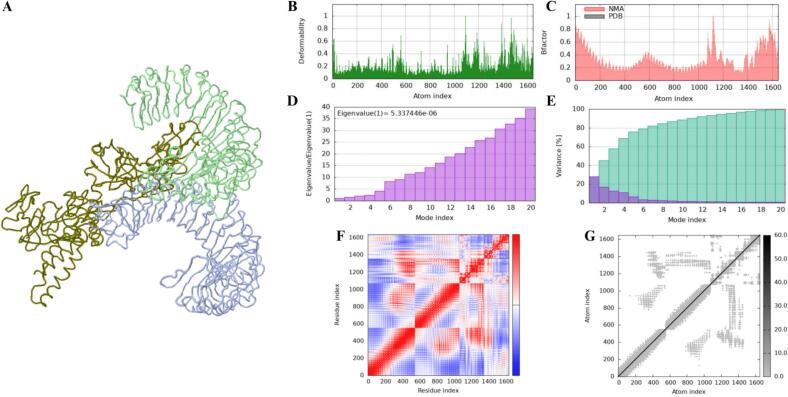
Normal mode analysis of the VEC1-TLR1/2 complex dynamics. (A) Complex mobility profiling via NMA. (B) Deformability plot to highlight the flexibility of specific residues along the molecular structure. (C) Comparison of B-factors between the NMA (red) and Protein Data Bank (PDB) data (gray), validating model dynamics. (D) Eigenvalue spectrum showing the first mode (lowest frequency) governing global motions. (E) Variance distribution across the first 20 normal modes, with individual (purple) and cumulative (green) contributions. (F) Covariance matrix of atomic motions: correlated (red), uncorrelated (white), and anti-correlated (blue). (G) Elastic network model depicting mechanical coupling through interatomic springs.

Molecular dynamics simulations, coupled with normal mode analysis (NMA), were performed on the VEC1 and TLR1/2 dimer docked complex, which demonstrated effective binding with the TLR complex (Fig. 8A). The B-factor values from the simulation indicated the root mean square (RMS) fluctuations of the atomic positions and so regions of high and low flexibility within the VEC1-TLR1/2 complex (Fig. 8B and C). The high level of flexibility within the construct likely contributes to the efficient binding of the construct to the TLR complex.

The eigenvalue plot (Fig. 8D) reflects the relative stiffness of the modes in the complex, where the eigenvalue of 5.3374447e^-06^ indicates the structural stability of the docked complex. In addition, the variance distribution highlights the overall stability and flexibility of the complex (Fig. 8E, purple – individual, green – cumulative). Our covariance matrix of atomic motions and elastic network model (Fig. 8F and 8G, respectively) also show areas of stiffness and flexibility, which further support VEC1′s structural stability and flexibility. Overall, VEC1 displays a suitable interaction with the TLR1-TLR2 complex with favorable dynamic properties, thereby enhancing its suitability as a TLR-targeting vaccine candidate (see Figs. S4 and S5 for VEC1 docked against TLR4 and 5, respectively).

### 5.12 Prediction of the secondary structure and surface accessibility of VEC1

**Fig. 9:**
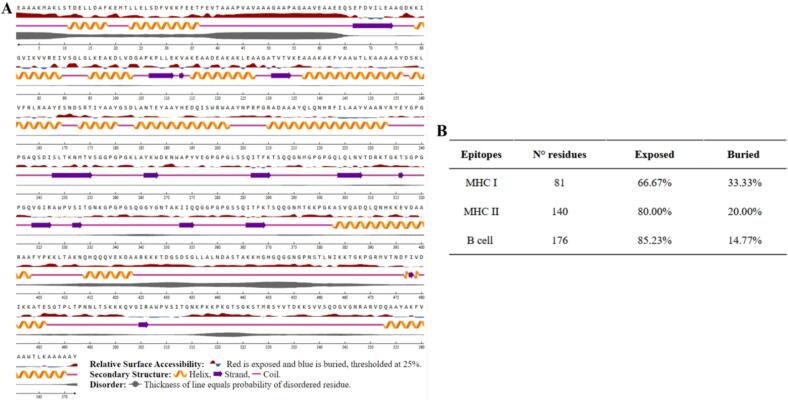
Structural and immunogenic features of VEC1. (A) Secondary structure mapping with α-helices (orange), β-strands (purple), and coils (pink). Surface accessibility of amino acid residues is coded (red indicating exposed and blue buried residues). Additionally, the gray line represents the disorder probability, where the thickness corresponds to the likelihood of disorder in the coil regions. (B) Epitope distribution showing B-cell, MHC I, and MHC II epitopes with the respective number and the percentage of exposed and buried residues.

The secondary structure analysis of our VEC1 vaccine revealed that a significant portion of its structure consisted of coiled regions, accounting for 56.29 % of its total (Fig. 9A). Additionally, 34.09 % and 9.82 % of VEC1 was composed of α-helix domains and β-strand domains, respectively (Fig. 9A). The solvent accessibility analysis showed that a substantial number of residues (66.67 %) in the MHC-I epitope cluster were exposed to the solvent, while 33.33 % remained buried within the structure. Similarly, the MHC-II epitope cluster had 80 % of residues exposed, with the remaining 20 % buried. Furthermore, for the B-cell epitopes, 85.22 % of residues were exposed, leaving 14.77 % buried (Fig. 9B). These findings provide valuable insights into the structural properties and potential immunogenicity of the VEC1 vaccine, indicating that its surface accessibility is optimized to elicit an effective immune response.

### 5.13 Discontinuous B cell epitopes

**Fig. 10:**
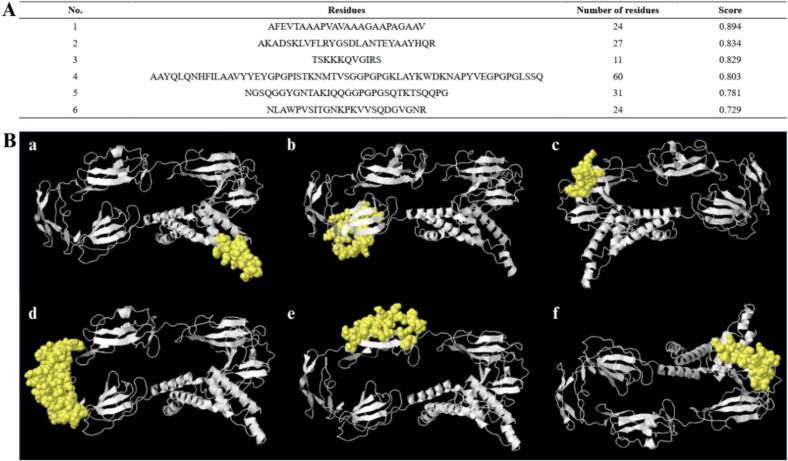
Structural predictions of conformational B-cell epitopes. (A) Epitope residues with position numbering and predicted confidence scores. (B) 3D structural mapping of six dominant epitopes (a-f), with predicted antigenic regions highlighted in yellow against the full protein structure (white).

The ElliPro online tool on the IEDB server identified six discontinuous B-cell epitopes, with scores ranging from 0.729 to 0.894. The amino acid residues forming these discontinuous epitopes, including the number of residues and their respective scores are detailed in Fig. 10A. The shortest predicted discontinuous B-cell epitope contained 11 residues, while the longest contains 60 residues. These epitopes were further analyzed, and their 3D structural representations are highlighted in yellow in Fig. 10B.

### 5.14 Simulations of immune responses to assess vaccine efficacy

**Fig. 11:**
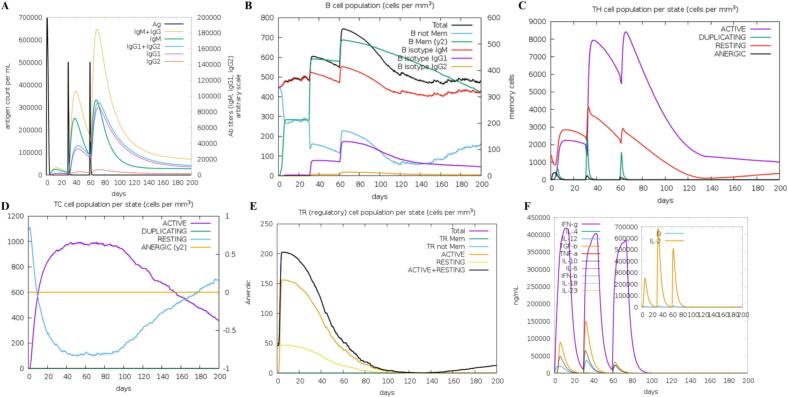
*In silico* simulation of the immune response after administration of the VEC1 vaccine as an antigen. (A) Level of antigens and immunoglobulins in response to the vaccine administered at different time periods. (B) Dynamics of B-cell populations, measured in cells per mm^3^. (C) T-helper cell populations. (D) Cytotoxic T-cell subsets: active (proliferating), resting (cells not exposed to the antigens), and anergic (tolerant). (E) Regulatory T-cell (Treg) compartments showing memory/effector differentiation. (F) Levels of cytokines after injection. The main plot displays overall cytokine levels, while the inset shows IL-2 levels and the Simpson index (D), which measures T-cell clonotypic diversity. A higher Simpson index indicates greater diversity.

Through immune simulations, we modeled the systemic exposure of antigens within lymphoid tissue throughout the three-dose VEC1 vaccination schedule. The vaccine was initially injected at time zero, followed by two booster doses at 30 and 60 days after the initial injection. The primary immune response was characterized by elevated IgM levels, indicating initial immune activation. The secondary and tertiary responses demonstrated a stronger reaction, as evidenced by significantly increased levels of IgM + IgG, IgG1 + IgG2, and IgG1 antibodies, along with a faster decline in antigen concentration (Fig. 11A).

Elevated B cell activation was observed, particularly with increased levels of the B-cells (y2) and IgM, accompanied by prominent memory cell development (Fig. 11B). The expanded populations of helper T cells and cytotoxic T cells further supported the establishment of immunological memory, suggesting the vaccine's potential to elicit long-lasting immunity (Fig. 11C, D). Significant levels of T regulatory cells (Treg cells) were observed following VEC1 exposure, but these levels subsequently declined after a few days of antigen exposure (Fig. 11E). Furthermore, the VEC1 vaccine was shown to induce the production of key cytokines, including IFN-γ and IL-2 (Fig. 11F).

### 5.15 Human similarity and autoimmunity risk assessment

A pBLAST search was conducted using the vaccine construct against the human proteome. The analysis returned no hits, indicating that the designed vaccine construct does not share detectable homology with any known human proteins. This finding suggests that the vaccine is unlikely to trigger any undesired immune response due to molecular mimicry, supporting its potential for safe clinical application.

### 5.16 Codon adaptation of the final vaccine candidate

**Fig. 12:**
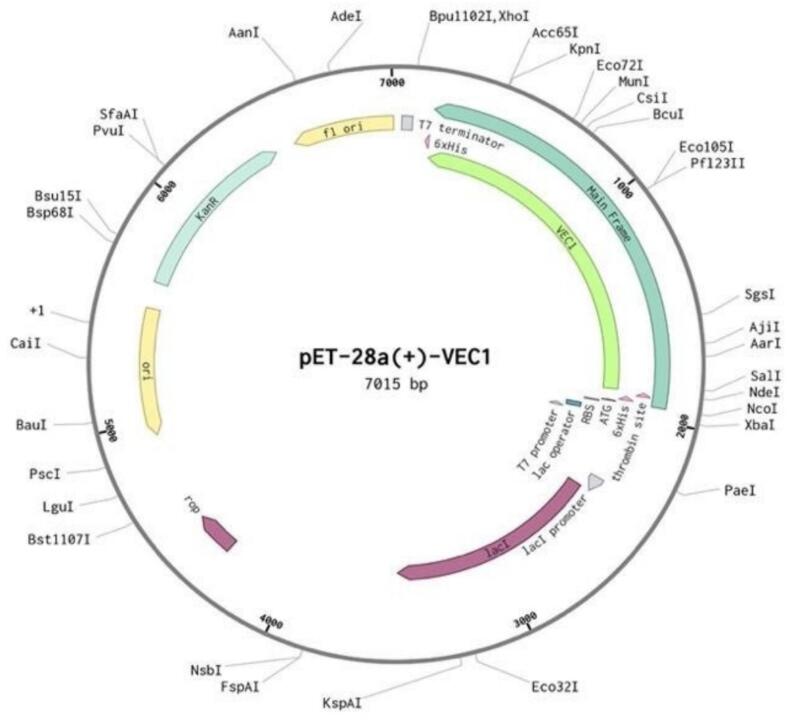
*In silico* design of the VEC-1 vaccine for expression in an *E. coli* system. Schematic representation of the vaccine sequence (light green) cloned into pET28a (+) vector using restriction sites. The expression cassette includes N-terminal His6-Tag and thrombin cleavage sites.

The codons of the VEC1 construct were optimized based on the codon usage bias of *E. coli* K12, using the Benchling webserver (https://www.benchling.com/). The final insert had a length of 1,731 base pairs, a GC content of 54 %, and a Codon Adaptation Index of 0.82. These parameters indicated a high probability of efficient vaccine expression in the *E. coli* K12 strain. The optimized sequence was cloned between the NdeI and XhoI restriction sites on the pET-28a expression vector, resulting in a construct with a total length of 7,015 base pairs (Fig. 12).

## 6 Discussion

Antimicrobial resistance is recognized as one of the world's most critical public health challenges.^35^, ^109^, ^110^ Vaccines represent a promising preventative approach, as they have significant potential to reduce the spread of highly pathogenic bacteria and minimize the reliance on antibiotics, which are the primary cause of antimicrobial resistance.^37^, ^39^, ^111^ In this context, we designed a multi-epitope-based vaccine against *Enterobacter cloacae* strains, an opportunistic pathogen that commonly causes nosocomial infections.^112^

Developing a vaccine candidate requires analyzing the sequences of core proteins that are conserved across all proteomes, as these represent promising targets for the creation of broad-spectrum antigenic candidates.^113^, ^114^ We conducted a subtractive analysis of twenty-one *E. cloacae* proteomes, and from the resulting conserved core proteome, we identified nine proteins exhibiting profiles indicative of essentiality, virulence, and resistance. These proteins were determined to be non-homologous to proteins in both humans and the gut microbiota, antigenic, and harboring extracellular domains or outer membrane localization. Essential proteins play a critical role in supporting the pathogen's growth, survival, and reproduction within its host, as well as performing key functional capabilities, meaning these genes cannot be easily discarded.^33^, ^115^ In addition to finding essential proteins, investigating virulence determinants and surface or secreted proteins is of particular interest for vaccine construction, given their capacity to interact with the host's immune system and elicit an immune response.^33^, ^102^ The antigenic properties of the protein allow it to be recognized by the immune system, eliciting a protective immune response in the host.^116^ Furthermore, screening for proteins that are non-homologous to both human and human gut microbiota can provide specificity to the pathogen. This reduces the chance of eliciting an autoimmune response in the host, while simultaneously protecting the symbiotic environment of the gut flora.^117^

Among the nine selected proteins, four were Curli proteins: the Curli major subunit CsgA, the Curli minor subunit CsgB, the Curli assembly chaperone CsgC and the Curli production assembly/transport protein CsgF. Curli form protein-based amyloid nanofibers that constitute the major component of the extracellular matrix in Gram-negative bacterial biofilms, endowing the bacteria with adhesive and invasive properties.^118^, ^119^ Five other proteins were identified and met our criteria. SfmF is a putative fimbrial protein found in the bacterium *E. coli* K-12 and may permit adhesion to various surfaces in specific environmental niches.^120^ FimH is a fimbrial adhesin that acts as a virulence factor in *E. coli*-induced urinary tract infections by promoting attachment to mannosylated glycoproteins on the bladder epithelial lining.^121^ Notably, FimH is a particularly promising vaccine candidate, as it has already progressed to phase II clinical trials for the prevention of recurrent urinary tract infections, utilizing a TLR4 agonist as an adjuvant.^122^ The heat shock protein (HslJ), a putative outer membrane protein, has been found to confer novobiocin resistance in *E. coli* and participates in numerous regulatory pathways essential for cellular functions at standard growth temperatures.^123^, ^124^ The protein MdtQ, detected in the outer membrane vesicles of carbapenem-resistant *Klebsiella pneumoniae*, plays a role in conferring multi-drug resistance.^125^ Finally, KdgM, a protein belonging to the oligogalacturonate-specific porin family, was identified as playing a pivotal role in the cellular uptake of acidic oligosaccharides.^126^

Despite the medical significance of *E. cloacae*, current research employing reverse vaccinology and subtractive genomics to identify potential vaccine targets against this pathogen is limited. However, some studies have made important contributions in this area. Al-Megrin et al. (2022) identified two proteins, the phosphoporin protein PhoE and a putative outer membrane porin protein, which were utilized to design a multi-epitope peptide vaccine against *E. cloacae.*^127^ In addition, Ismail et al. (2021) prioritized four target proteins, including the outer membrane usher protein LpfC, the putative outer membrane protein A OmpA, the putative outer membrane protein FimD, and an arginine transporter, as potential *E. cloacae* vaccine candidates.^128^ Furthermore, Bolourchi et al. (2022) recognized two distinct proteins, the TonB-dependent siderophore receptor and the YjbH domain-containing protein, as novel putative immunogenic targets against clinical isolates of the *E. cloacae* complex.^129^ More recently, Alhassan (2024) identified four proteins, the flagellar hook-associated protein FlgL, the TonB-dependent siderophore receptor, the porin OmpA, and the flagellar basal body rod protein FlgB, to construct a multi-epitope vaccine against the *E. cloacae* complex.^130^ Notably, the vaccine developed in our study incorporates different proteins that were identified in previous studies, thereby diminishing the existing knowledge gap in this area.

One strategy for vaccine design is to utilize multiple antigens, as these can induce a more comprehensive and robust immune response compared to single-component vaccines. Furthermore, incorporating multiple highly immunogenic antigens in vaccine design can help mitigate the impact of antigenic variation, which can occur when relying on a single antigen.^131^ In this way, we identified B cell, helper T cell, and cytotoxic T cell epitopes derived from our selected conserved proteins. The use of conserved core protein epitopes is expected to maintain efficacy against emerging variant strains. Here, mutations in the conserved regions are unlikely to occur due to the detrimental impact and fitness burden they would impose on the bacteria.^132^ The selected epitopes induced an antigenic response without triggering allergic reactions and exhibited favorable properties, such as hydrophilicity and non-toxicity.^133^

To ensure broad HLA coverage, the selected MHC-I and MHC-II epitopes were docked against a panel of relevant HLA variants. The epitopes with the best average docking scores, as well as B-cell epitopes, were utilized in vaccine construction. The vaccine design incorporated a combination of selected B-cell, helper T-cell, and cytotoxic T-cell epitopes, which were connected by AAY, GPGPG, and KK linkers. The alpha helix-forming linkers featuring the EAAAK sequence were added to the C- and N-terminals of the adjuvants.^134^ These linker sequences not only serve to physically separate the different peptide components, but they can also play a role in enhancing the effectiveness, stability, and immunogenic properties of the vaccine constructs.^83^, ^135^ Meanwhile, the AAY linkers support the formation of epitopes in a natural configuration and prevent the creation of junctional epitopes, as well as being used by mammalian proteasomes to cleave the target into separate epitopes.^99^ In contrast, the GPGPG linker stimulates robust T helper lymphocyte responses and preserves the epitope’s structure, whereas the KK linker serves as a cleavage site for the lysosomal protease, which is crucial for MHC class II-mediated antigen presentation.^136^ Adding to this, the EAAAK linker, a rigid linker with helix-forming properties, was selected to enhance the immunogenic properties of the construct.^137^, ^138^ The PADRE sequence was incorporated into the vaccine model to enhance the stability of the construct and optimize the efficacy and potency of the vaccine subunits, leading to improved cytotoxic T lymphocyte responses.^139^ Additionally, the vaccine designs incorporated adjuvants derived from the pathogens themselves, including FliC flagellin, HBHA protein, the conserved HBHA sequence, or the 50S ribosomal protein L7/L12. FliC stimulates the immune system through Toll-like receptor 5,^140^ while the 50S ribosomal protein L7/L12 and HBHA sequence have the ability to interact with Toll-like receptor 4.^141^ These adjuvants can therefore elicit more targeted and effective immune responses.^135^

Our analyses resulted in the development of four vaccine candidates: VEC1, VEC2, VEC3, and VEC4. All vaccines were shown to possess desirable properties, including molecular weights, high isoelectric points, non-allergenic nature, physical stability, hydrophilic character, and solubility. More importantly, these candidates exhibited high antigenicity and the capacity to elicit a robust immune response. The study also evaluated the susceptibility of the vaccine's epitopes, separated by linkers, to degradation by proteasomal and cathepsin-specific peptidase activity. Our findings indicated that the selected linkers and their arrangement were appropriate, enabling the produced epitopes to be effectively presented and processed by the host's immune system, eliciting both humoral and cellular immune responses.^43^

Vaccines play a crucial prophylactic role and require extensive coverage to address future challenges posed by deadly infections faced by the healthcare community. Consequently, a multi-epitope vaccine needs to be suitable across the global variation in HLA alleles to meet the needs of different ethnic groups.^142^ Our vaccines are predicted to cover 92.6 % of the global population, where 90 % coverage has been shown previously to be sufficiently effective to be used around the world.^142^, ^143^, ^144^, ^145^

All four vaccine candidates underwent 3D structural modeling and refinement to offer valuable insights into their protein–ligand interactions.^146^ All models were validated to accurately compare the unrefined and refined structures.^68^ Through our modeling, we confirmed that most residues of interest in our vaccine structures were in favorable regions, indicating that the final structures were of acceptable quality.

Toll-like receptors play a pivotal role in bridging innate and adaptive immune responses by modulating antigen-presenting cell activation and regulating the production of critical cytokines.^147^ The TLR1/2 complex plays a central role in immune responses, as it is crucial for microbial infection, dendritic cell maturation, T helper cell differentiation, and regulation of signaling pathways.^148^ The TLR2 is also particularly known for its involvement in recognizing a wide range of microbial components, partly due to its synergistic interactions with other TLRs, such as TLR1, TLR6, and, in some cases, TLR4.^149^ Furthermore, TLR1/2 agonists are among the most potent adjuvants that enhance vaccine responses in the elderly population.^150^ The binding patterns and stability of the four vaccine candidates with Toll-like receptors were assessed through molecular docking and molecular dynamics simulations. VEC1 exhibited the strongest binding affinity with TLR1/2, TLR4, and TLR5 when compared to VEC2-4. Ligand binding induces heterodimerization of TLR1 and TLR2 ectodomains, leading to structural rearrangement and downstream signalling.^151^ Here, the VEC1-TLR1/2 complex had a higher number of interacting residues using polar contacts and fewer electrostatic clashes, particularly at the C-terminal domains. These interactions are crucial in determining protein binding constants, even at relatively long ranges, as polar interactions between charged residues confer specificity in protein–protein interactions.^152^ This suggests that the interactions between VEC1 and amino acids of TLR1/2 form highly stable complexes that would activate cells and downstream signals. This stable interaction was further confirmed with molecular dynamics simulations, as the VEC1-TLR1/2 complex presented the lowest eigenvalue and stiffer regions compared to the TLR4 and TLR5 complexes, indicating a more stable and flexible molecular motion.^153^ Our findings align with those of Zhu et al. (2025), who demonstrated the strong binding affinity of multi-epitope vaccines targeting *Pseudomonas aeruginosa* to Toll-like receptors 1 and 2.^148^

Next, the secondary structure and surface accessibility of VEC1 were predicted, as these are crucial in an effective vaccine.^36^ The VEC1 vaccine was found to have a high proportion of coiled residues (56.29 %), followed by α-helical (34.09 %) and β-strand (9.62 %) domains. The α-helical coiled-coil motifs are promising candidate antigens for vaccine development, as they are highly immunogenic and have demonstrated protective effects in numerous in vitro functional assays.^135^, ^154^ Our data indicated that the majority of MHC-I, MHC-II, and B-cell epitopes were exposed, suggesting potential regions for epitope recognition and vaccine stability.^155^ The spatial arrangement of discontinuous or conformational B-cell epitopes is fundamental to vaccine design and is influenced by protein folding.^36^ The identification of discontinuous B-cell epitopes and their arrangement within our VEC1 vaccine construct hints that they may have the potential to interact with antibodies and exhibit structural flexibility, making VEC1 a favorable candidate.

The immune simulation showed the VEC-1 vaccine generated elevated levels of cytotoxic and helper T cells, as well as IFN-γ. Our data align with reports suggesting these parameters are critical for a robust humoral and adaptive immune response.^83^, ^137^, ^156^, ^157^ Interestingly, prior studies have found that vaccines evaluated through *in silico* immunological simulations not only demonstrate the ability to elicit a robust immunogenic response, but also the capacity to produce antibodies *in vivo.*^158^ Furthermore, the nucleotide sequence of VEC1 was optimized for transcriptional and translational efficiency within the *E. coli* strain K12 by considering its GC content and codon adaptation index (CAI).^68^ The final construct was designed with NdeI and XhoI restriction sites, enabling its cloning into the pET28a + expression vector, which suggests its suitability for *in vitro* testing.

In conclusion, this study employed various computational techniques to develop a potential vaccine candidate, VEC1, against the *E. cloacae* pathogen. The data demonstrated that the VEC1 vaccine possesses favorable structural and physicochemical properties and has the potential to elicit both humoral and cell-based immune responses. Furthermore, it was predicted that VEC1 can be readily expressed in *E. coli* strains. While the application of immunoinformatics approaches proved valuable in guiding the experimental studies for the vaccine, our study had some limitations. For instance, the available computational tools were unable to identify carbohydrate-based antigens, which can be common protective antigens.^159^ These types of analyses often prioritize highly expressed antigens or those with distinct signaling peptides while overlooking less abundant, but possibly equally effective, antigens.^142^ Consequently, other epitopes that still contribute to the immune response may have been underexplored. The immune response predicted by *in silico* simulations may not account for factors like pre-existing medical conditions and age that can influence an individual's immune response after receiving the vaccine in real-world settings.^83^ Experimental validation in animal models is critical to confirm immunogenicity, safety, and protective efficacy—bridging computational promise with real-world application.^160^ As a result, the proposed multi-epitope vaccine candidate will undergo extensive evaluation and validation in the next phase of studies. This will involve a detailed analysis of its ability to induce a robust and protective immune response, as well as a thorough assessment of its overall safety profile in relevant animal models. The findings from these studies are expected to contribute to the successful development and clinical application of the VEC1 vaccine against *E. cloacae* strains.

## CRediT authorship contribution statement

**Gabriela Guerrera Soares:** Writing – review & editing, Writing – original draft, Visualization, Validation, Methodology, Investigation. **Marcelo Silva Folhas Damas:** Writing – review & editing, Writing – original draft, Visualization, Validation, Methodology, Investigation. **Pedro Mendes Laprega:** Visualization, Validation, Methodology. **Rebecca Elizabeth Shilling:** Writing – review & editing, Writing – original draft, Visualization. **Eduarda Oliva Ribeiro Rangel:** Visualization, Methodology. **Louise Teixeira Cerdeira:** Visualization, Validation. **Murillo Rodrigo Petrucelli Homem:** Visualization, Validation. **André Pitondo-Silva:** Writing – review & editing, Validation. **Andrea Soares da Costa-Fuentes:** Writing – review & editing, Visualization. **Maria-Cristina da Silva Pranchevicius:** Writing – review & editing, Writing – original draft, Visualization, Validation, Supervision, Resources, Project administration, Funding acquisition, Formal analysis, Conceptualization.

## 7 Funding

The authors declare they received financial support for this research. The study was supported by the Fundação de Amparo à Pesquisa do Estado de São Paulo-Brazil (FAPESP grant 2022/16872-6, 2020/11964-4, to MCSP). It was also partially funded by the same organization as fellowships for GGS (FAPESP fellowship 2021/08423-4), MSFD (FAPESP fellowship 2018/24213-7), EORR (FAPESP fellowship 2022/08011-0), and PML (FAPESP fellowship 2021/00425-8). Additionally, the study was financed in part by the Coordenação de Aperfeiçoamento de Pessoal de Nível Superior – Brazil (CAPES- Finance code 001) as a fellowship for GGC.

## Declaration of competing interest

The authors declare the following financial interests/personal relationships which may be considered as potential competing interests: With the submission of this manuscript we would like to undertake that this manuscript has not been published, accepted for publication and that the authors have. not any competing interests. If there are other authors, they declare that they have no known competing financial interests or personal relationships that could have appeared to influence the work reported in this paper.
