# Supplementary material for: Alternative therapeutic approaches for combating multi-drug-resistant bacteria: Reverse vaccinology against Enterobacter cloacae
Source: J Genet Eng Biotechnol. 2025 Jun 17;23(3):100519. doi: 10.1016/j.jgeb.2025.100519 (PMC12210309; doi:10.1016/j.jgeb.2025.100519)
Supplement: Supplementary Data 1 [file mmc1.docx]

**SUPPLEMENTARY**

**TABLES**

**Table S1**. The clinical *Enterobacter cloacae* strains analyzed in this study.

| **Organism Strain ID** | **RefSeq Assembly** | **Biosample** | **Geographic Origin** | **Isolation Year** |
| --- | --- | --- | --- | --- |
| *Enterobacter cloacae* 1382* | GCF_905331265.2 | SAMEA8065834 | Spain: Barcelona | 2022 |
| *Enterobacter cloacae* FDAARGOS 1431 | GCF_019047105.1 | SAMN16357573 | Germany: Braunschweig | 2021 |
| *Enterobacter cloacae* CZ862 | GCF_018140965.1 | SAMN18753389 | Czech Republic: Prague | 2021 |
| *Enterobacter cloacae* EFN743 | GCF_023920605.1 | SAMN26208495 | Ghana | 2022 |
| *Enterobacter cloacae* PIMB10EC27 | GCF_002982195.1 | SAMN06448868 | Viet Nam | 2018 |
| *Enterobacter cloacae* isolate F | GCF_032349915.1 | SAMN37529177 | Canada: Ottawa | 2023 |
| *Enterobacter cloacae* CBG15936 | GCF_009707405.1 | SAMN13288306 | China: Guangzhou | 2019 |
| *Enterobacter cloacae* 14240244101 | GCF_016864115.1 | SAMN12999973 | Netherlands: Groningen | 2021 |
| *Enterobacter cloacae* 3143 | GCF_024917615.1 | SAMN28174020 | USA: Houston | 2022 |
| *Enterobacter cloacae* 2022CK-00409 | GCF_025426135.1 | SAMN29981189 | USA | 2022 |
| *Enterobacter cloacae* M12X01451 | GCF_002303275.1 | SAMN05831100 | Unknown | 2017 |
| *Enterobacter cloacae* ECl_1.VN | GCF_020540925.1 | SAMN22169090 | South Africa: Pretoria | 2021 |
| *Enterobacter cloacae* isolate B | GCF_032352615.1 | SAMN37529173 | Canada: Ottawa | 2023 |
| *Enterobacter cloacae* 99183042147 | GCF_031851625.1 | SAMN37298531 | France: Montpellier | 2023 |
| *Enterobacter cloacae* 53EVA | GCF_033802605.1 | SAMN38124868 | Singapore | 2023 |
| *Enterobacter cloacae* SD21 | GCF_023238665.1 | SAMN26686306 | China: Shandong | 2022 |
| *Enterobacter cloacae* Ecc8276_LB-HALD | GCF_030406505.2 | SAMN35795218 | Senegal: Dakar | 2023 |
| *Enterobacter cloacae* 12961-yvys | GCF_023023085.1 | SAMN21379556 | China | 2022 |
| *Enterobacter cloacae* Colony146 | GCF_019265085.1 | SAMN17833497 | Thailand | 2021 |
| *Enterobacter cloacae* Colony187 | GCF_019711235.1 | SAMN17833490 | Thailand: Uttaradit | 2021 |
| *Enterobacter cloacae* NH77 | GCF_006228165.1 | SAMN11866226 | Thailand: Chiang Mai | 2019 |

* Reference strain.

**FIGURES**


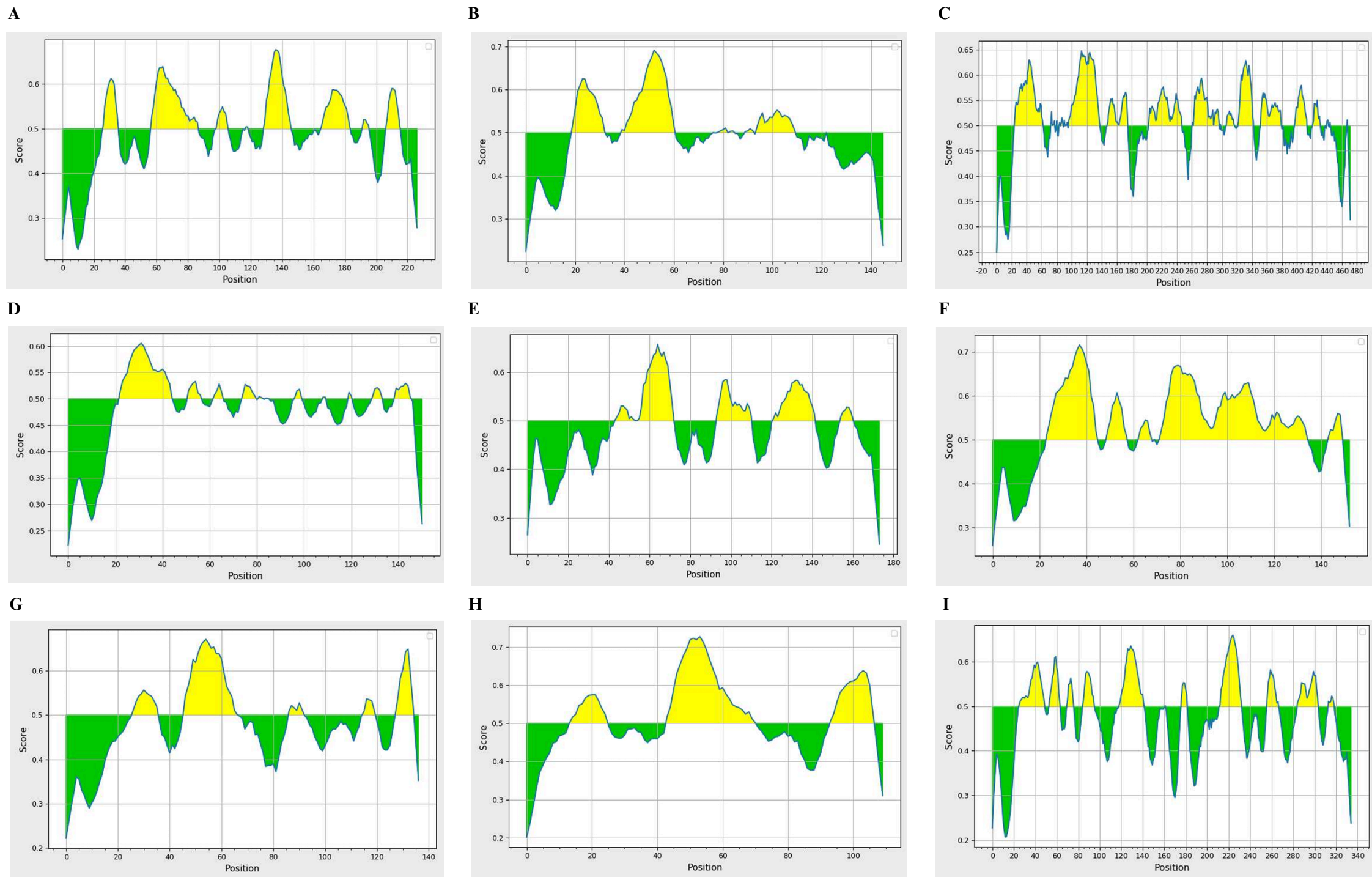


**Figure S1****.** Prediction of linear B cell epitopes using the IEDB Kolaskar and Tongaonkar antigenicity scale. (A) Oligogalacturonate-specific porin KdgM family protein, (**B**) Heat shock protein HslJ, (**C**) Multi-drug resistance outer membrane protein MdtQ, (**D**) Curli minor subunit CsgB, (**E**) putative fimbrial assembly protein SfmF, (**F**) Curli major subunit CsgA, (**G**) Curli production assembly/transport protein CsgF, (**H**) Curli assembly chaperone CsgC, and (**I**) Type 1 fimbria D-mannose specific adhesin FimH. Antigenic potential was assessed using BepiPred (threshold > 0.5). Yellow - immunogenic epitopes, green - non-antigenic sequences.


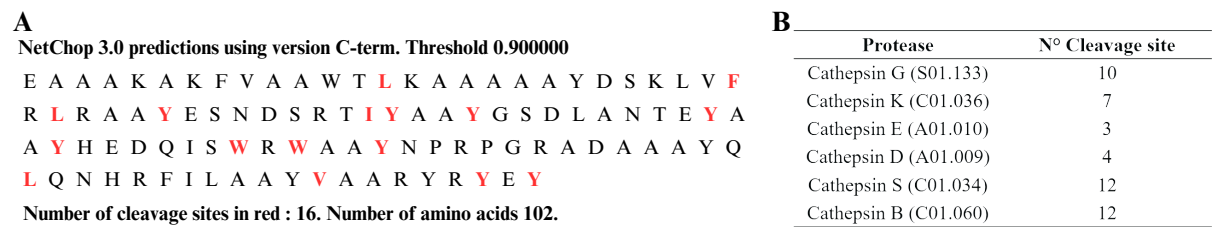


**Figure S2.** Epitope processing predictions for vaccine candidates. (A) MHC-I epitopes generated through proteasomal cleavage, with cleavage sites indicated in red within the amino acid sequence. (B) MHC-II epitopes produced via cleavage by cathepsins, including the number of cleavage sites for each protease.

**
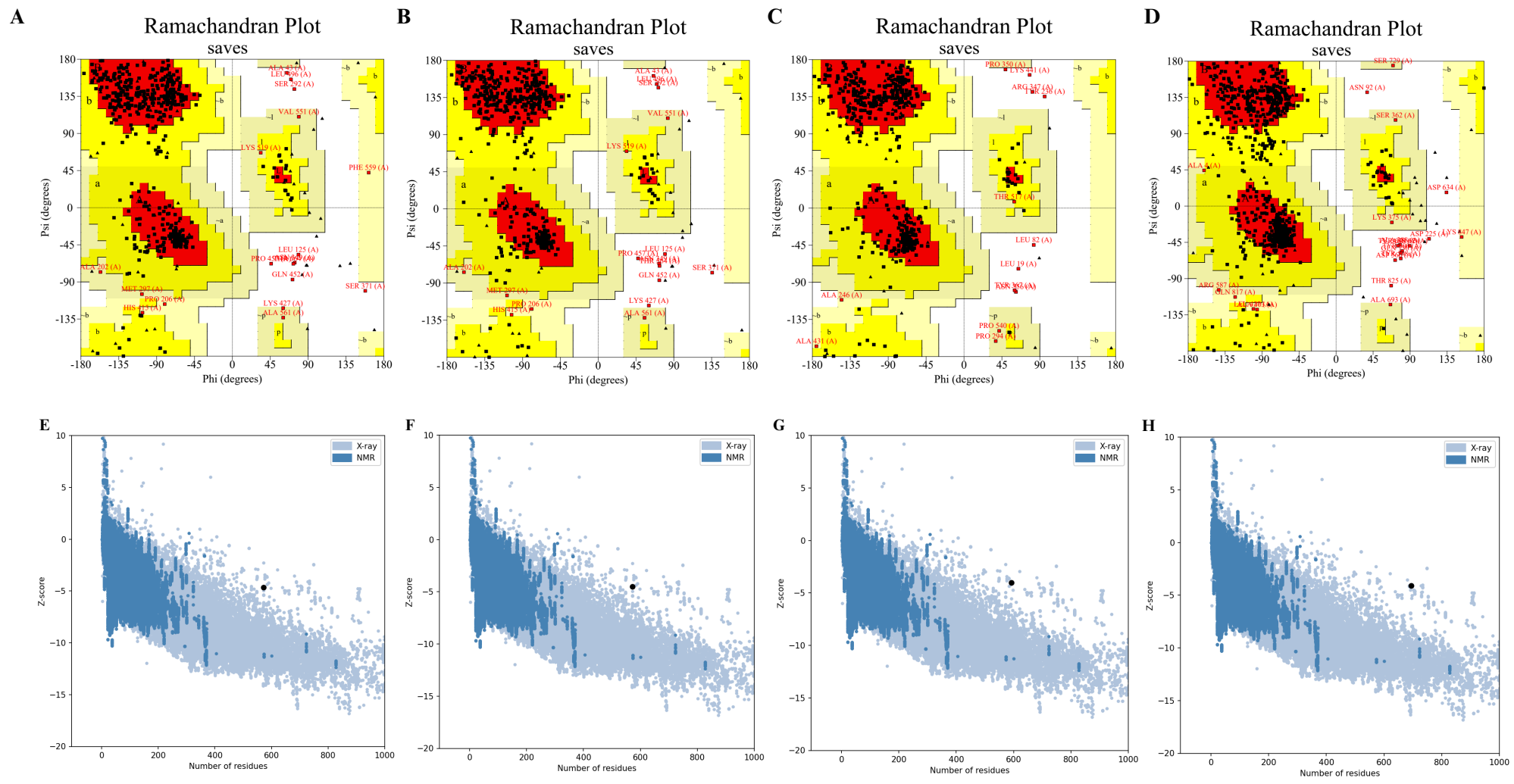
**

**Figure S3.** Structural validation of vaccine constructs**.** (**A-D**) Ramachandran plots for refined VEC1-4 models showing residue distribution: most favored regions (red), allowed regions (yellow), and disallowed regions (white). (**E-H**) ProSA-web Z-scores (black dots) for each construct.


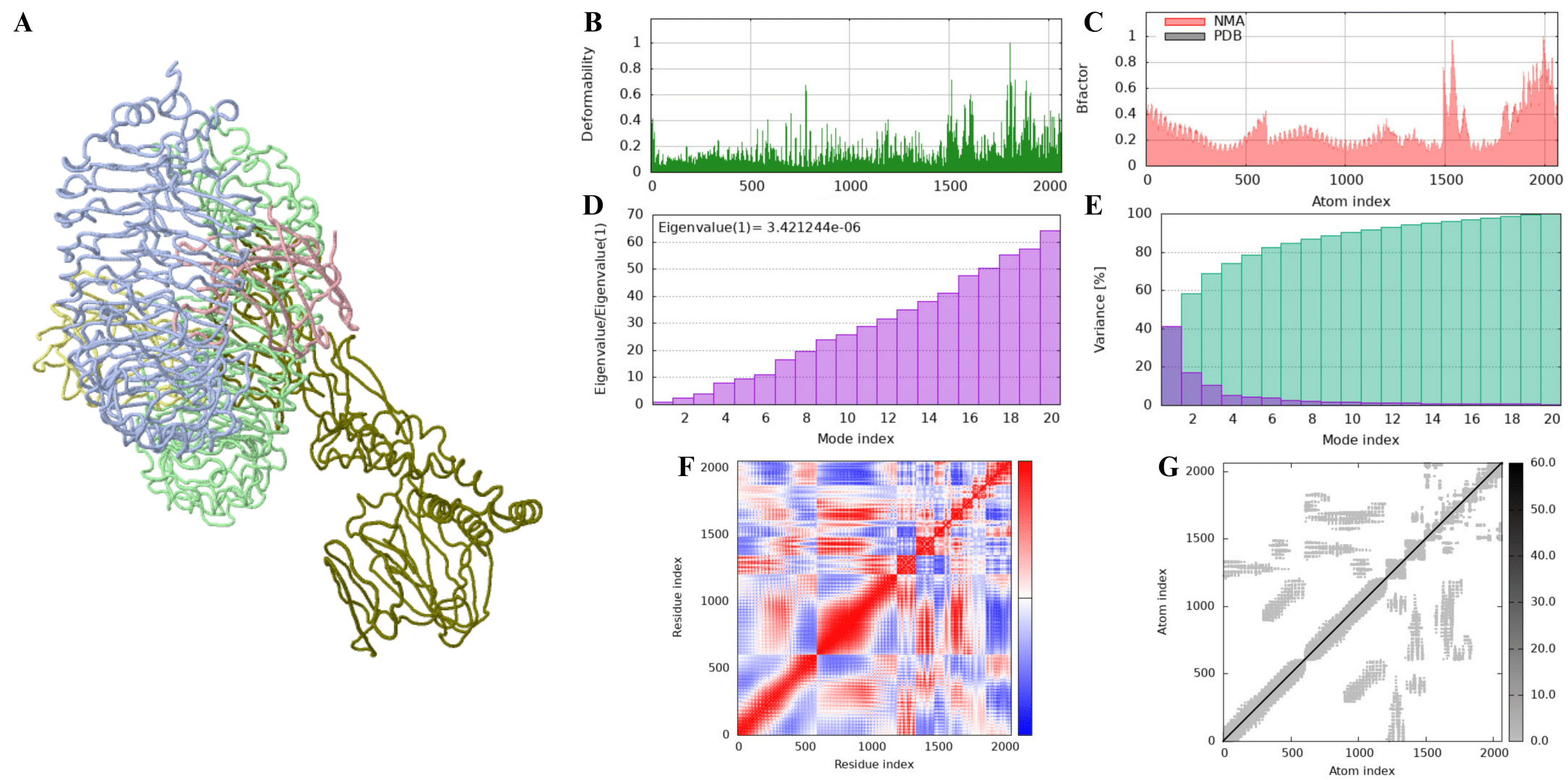


**Figure S4.** Normal mode analysis of VEC1-TLR4 complex dynamics. (A) Complex mobility profiling via NMA. (B) Deformability plot to highlight the flexibility of specific residues along the molecular structure. (C) Comparison of B-factors between the NMA (red) and Protein Data Bank (PDB) data (gray), validating model dynamics. (D) Eigenvalue spectrum showing the first mode (lowest frequency) governing global motions. (E) Variance distribution across the first 20 normal modes, with individual (purple) and cumulative (green) contributions. (F) Covariance matrix of atomic motions: correlated (red), uncorrelated (white), and anti-correlated (blue). (G) Elastic network model depicting mechanical coupling through interatomic springs.


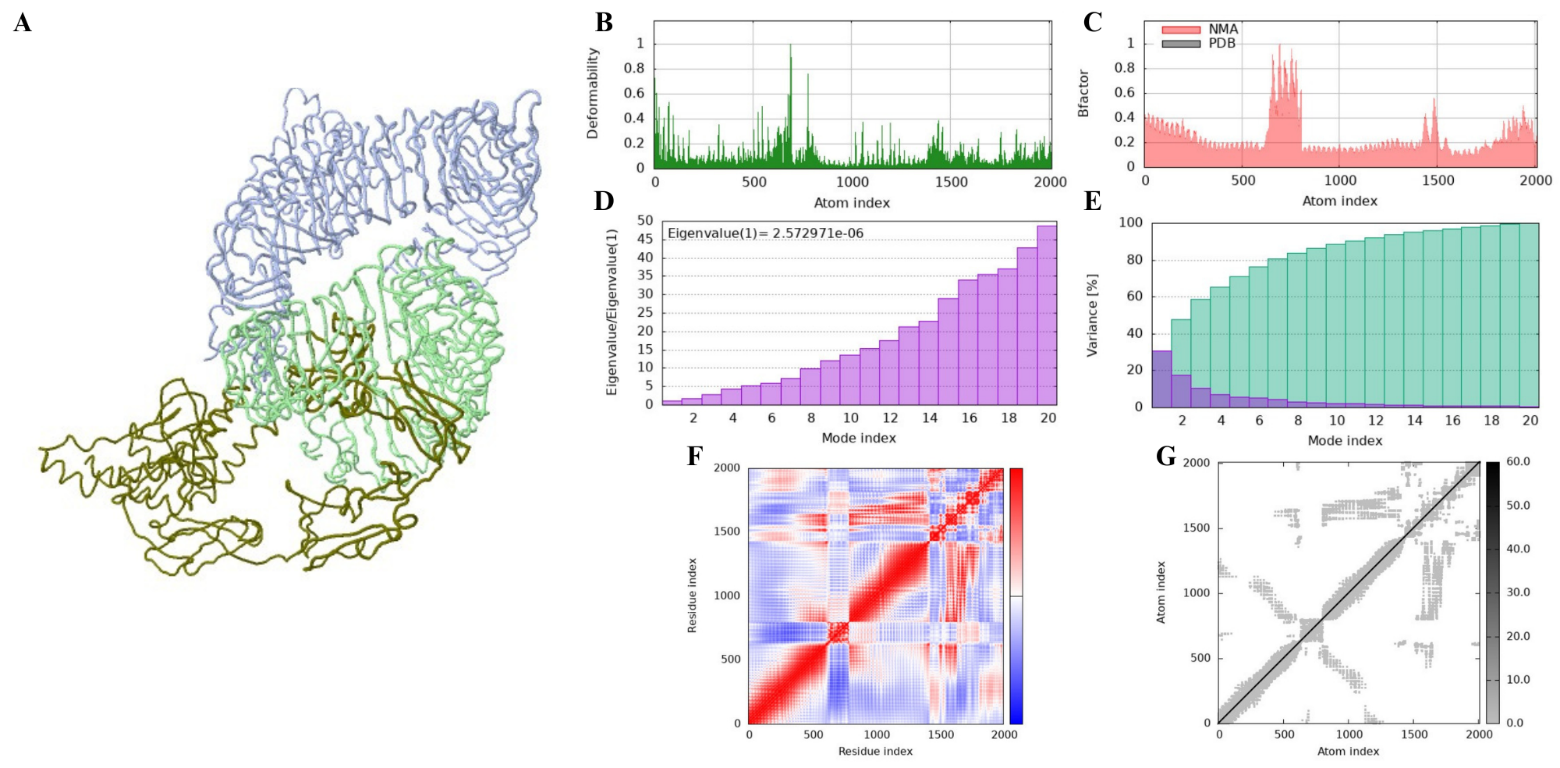


**Figure S5.** Normal mode analysis of VEC1-TLR5 complex dynamics. (A) Complex mobility profiling via NMA. (B) Deformability plot to highlight the flexibility of specific residues along the molecular structure. (C) Comparison of B-factors between the NMA (red) and Protein Data Bank (PDB) data (gray), validating model dynamics. (D) Eigenvalue spectrum showing the first mode (lowest frequency) governing global motions. (E) Variance distribution across the first 20 normal modes, with individual (purple) and cumulative (green) contributions. (F) Covariance matrix of atomic motions: correlated (red), uncorrelated (white), and anti-correlated (blue). (G) Elastic network model depicting mechanical coupling through interatomic springs.
